# Connectome embedding in multidimensional graph spaces

**DOI:** 10.1162/netn_a_00393

**Published:** 2024-12-10

**Authors:** Mathieu Mach, Enrico Amico, Raphaël Liégeois, Maria Giulia Preti, Alessandra Griffa, Dimitri Van De Ville, Mangor Pedersen

**Affiliations:** Neuro-X Institute, Ecole Polytechnique Fédérale De Lausanne (EPFL), Geneva, Switzerland; Department of Radiology and Medical Informatics, University of Geneva (UNIGE), Geneva, Switzerland; CIBM Center for Biomedical Imaging, Lausanne, Switzerland; Leenaards Memory Center, Lausanne University Hospital and University of Lausanne, Lausanne, Switzerland; Department of Psychology and Neuroscience, Auckland University of Technology, Auckland, New Zealand

**Keywords:** Connectome, Graph space, Network analysis, Single brain region, Global brain, Distance

## Abstract

Connectomes’ topological organization can be quantified using graph theory. Here, we investigated brain networks in higher dimensional spaces defined by up to 10 graph theoretic nodal properties. These properties assign a score to nodes, reflecting their meaning in the network. Using 100 healthy unrelated subjects from the Human Connectome Project, we generated various connectomes (structural/functional, binary/weighted). We observed that nodal properties are correlated (i.e., they carry similar information) at whole-brain and subnetwork level. We conducted an exploratory machine learning analysis to test whether high-dimensional network information differs between sensory and association areas. Brain regions of sensory and association networks were classified with an 80–86% accuracy in a 10-dimensional (10D) space. We observed the largest gain in machine learning accuracy going from a 2D to 3D space, with a plateauing accuracy toward 10D space, and nonlinear Gaussian kernels outperformed linear kernels. Finally, we quantified the Euclidean distance between nodes in a 10D graph space. The multidimensional Euclidean distance was highest across subjects in the default mode network (in structural networks) and frontoparietal and temporal lobe areas (in functional networks). To conclude, we propose a new framework for quantifying network features in high-dimensional spaces that may reveal new network properties of the brain.

## Author Summary

Nodal properties are of particular importance when investigating patterns in brain networks. Nodal information is usually studied by comparing a few nodal measurements (up to three), resulting in analyses in three-dimensional spaces, at maximum. We offer a new framework to extend these approaches by defining new, up to 10-dimensional, mathematical spaces, called graph spaces, built using up to 10 nodal properties. We show that correlations between nodal properties express differences regarding connectome models (structural/functional, binary/weighted) and brain subnetworks. We provide early application and quantification of machine learning in graph spaces of dimensions 2 to 10, as well as a quantification of single brain regions, and global connectome, Euclidean distance in a 10-dimensional graph space. This provides new tools to quantify network features in high-dimensional spaces.

## INTRODUCTION

Network theory has become an emerging avenue of investigation in science (Das et al., 2018; Du et al., 2015; Jordán et al., 2007; Kleinberg & Lawrence, 2001; Park, 2003; Pedersen et al., 2020). Network analysis is particularly relevant in neuroscience since the brain and its neurons comprise complex and multiscale interconnected networks (Avena-Koenigsberger et al., 2015; Bullmore & Sporns, 2009; Farahani et al., 2019; Fornito et al., 2016; Goñi et al., 2013; Simas et al., 2015; Sporns, 2016; Stam, 2014; Wang et al., 2010). A better comprehension of brain networks is a critical element in searching not only for simple and noninvasive diagnostic markers of neuropsychiatric and neurological diseases (Bassett & Bullmore, 2009; Zhao et al., 2019) but also for the general understanding of how the different brain structures interact (Sporns, 2018; Yeo et al., 2011). An essential idea of network theory is the concept of node-level invariants, called in this study as nodal properties, which are nodal scores reflecting the nodes’ “importance” or topological role in the whole network. In graph theoretical terms, nodal properties can be divided into integration measures (e.g., centralities) and segregation measures (e.g., clustering coefficient). Using graph nodal properties, we can also build a ranking of nodes and compare different nodes.

In computational and clinical neurosciences, many studies have used nodal properties at different scales to characterize the brain organization in healthy and diseased populations (Bassett & Bullmore, 2009; Stam, 2014; Sun et al., 2019; van den Heuvel & Sporns, 2019), with popular nodal property measures being the degree, betweenness, closeness, and eigenvector centrality (Joyce et al., 2010; Kuhnert et al., 2012; Mantzaris et al., 2013; Zuo et al., 2012). Moreover, nodal properties can be combined and considered jointly within multidimensional spaces. For example, Joyce and colleagues (Joyce et al., 2010) defined a new nodal property called leverage centrality and compared it with three well-known nodal properties in connectomes (degree, betweenness, and eigenvector centrality). To this end, they defined two-dimensional (2D) and three-dimensional (3D) spaces, where the different nodal properties represent the spaces’ dimensions and the single brain regions are points living in these spaces. Zuo and colleagues (Zuo et al., 2012) used voxel-level nodal property scatterplots to investigate the relationship between different invariants of brain functional connectomes. Nonetheless, most work on graph nodal properties applied to connectomes has been done in 1D, 2D, or 3D spaces, that is, considering a few nodal properties at a time.

Our current study aims to extend these works by investigating brain connectomes within high-dimensional nodal properties’ spaces. To this end, we considered up to 10 graph nodal properties and used them to define multidimensional spaces within which we characterized internodal and intersubject connectomes’ distances. Our work proposes a new way to use graph nodal properties by creating Euclidean spaces, where each axis represents a graph nodal property. Each node (brain region) has a precise position in these spaces, defined by its nodal scores that make a set of coordinates.

Three main analyses were performed in this study. First, to understand similarities and dissimilarities between different nodal properties, we conducted a correlation analysis between nodal properties, both at whole-brain and functional subnetworks’ levels. Second, we explored the multidimensional graph spaces derived from up to 10 nodal properties using machine learning (ML) to test whether brain regions belonging to different brain circuits can be differentiated in such spaces and provide early results on how much information is gained when using higher spaces. Finally, we characterized each brain region based on its interindividual mean distances in multidimensional graph spaces. This analysis allowed us to identify the brain regions yielding the largest interindividual variability in a multidimensional graph space.

## RESULTS

**Figure 1.**
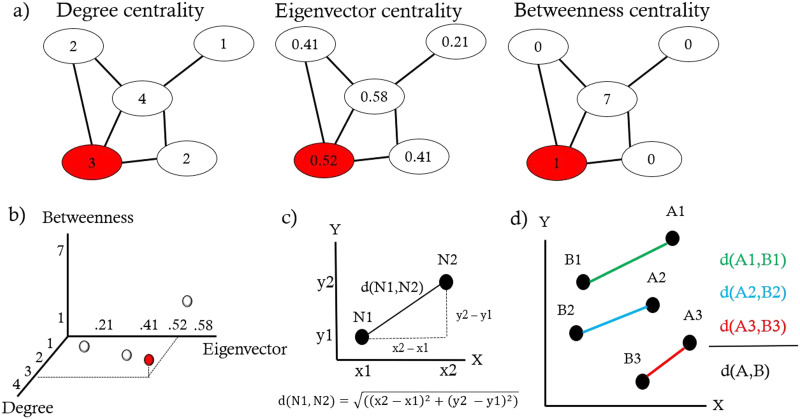
Schematic of graph nodal properties in Euclidean space and node distance. (A) Nodal degree, eigenvector, and betweenness centrality nodal properties computed on the same toy graph. (B) Illustration of the 3D metric space (3D graph space) built from the above-listed nodal properties (degree, eigenvector, and betweenness centrality). Each dot represents a node of the toy graph in (A). As an example, the red dot corresponds to the red node. (C) Euclidean distance *d* between two graph nodes embedded in a 2D space. (D) Euclidean distances between homologous brain regions (regions 1, 2, and 3) from different subjects (A and B), and the average distance between the two subjects across homologous brain regions.

Our approach comprises three steps and is schematized in Figure 1. First, each connectome’s different nodal properties are computed (Figure 1A). The 10 nodal properties considered in this work are listed and explained in more detail in the Graph Invariants section. The rationale for investigating 10 nodal properties is to understand which additional information about the human connectomes we can gain compared with considering smaller nodal properties’ subsets. It also allows exploring different combinations of invariants in lower dimensional spaces (2D and 3D, for example) to study connectomes. The next step is to interpret each computed nodal property as an axis of a multidimensional Euclidean space, as well as the nodal property scores of each brain region (network node) as coordinates in such space (Figure 1B). This results in embedding each node in a new multidimensional space that we name “graph space.” The value of this embedding is that brain regions are placed in the graph space according to their properties concerning the whole-brain network and can be easily compared between each other and across different subjects. The final step is to explore this new space. We propose to compute pairwise distances between brain regions in the graph space. We used the Euclidean distance since we are working in an Euclidean space and it can be very easily defined and calculated in a space of any dimension (Figure 1C). The Euclidean distance can also be used to compare connectomes from different subjects at multiple scales (Figure 1D), for example, considering a single brain region across subjects or an average global distance.

### Graph Nodal Properties Are Correlated at Whole-Brain and Subnetwork Levels

We derived group-representative connectomes from structural, diffusion, and resting-state functional magnetic resonance imaging (fMRI) data of 100 healthy subjects of the Human Connectome Project (Van Essen et al., 2013), each one composed of 219 nodes (brain regions). The 219 regions were grouped into nine resting-state networks (RSNs) (Yeo et al., 2011). Multiple connectome models were considered: binary structural and functional (SCBIN and FCBIN), as well as weighted structural (length-based) and functional (Length-SCWEI and FCWEI). Further details about constructing these connectomes can be found in the Connectomes section.

**Figure 2.**
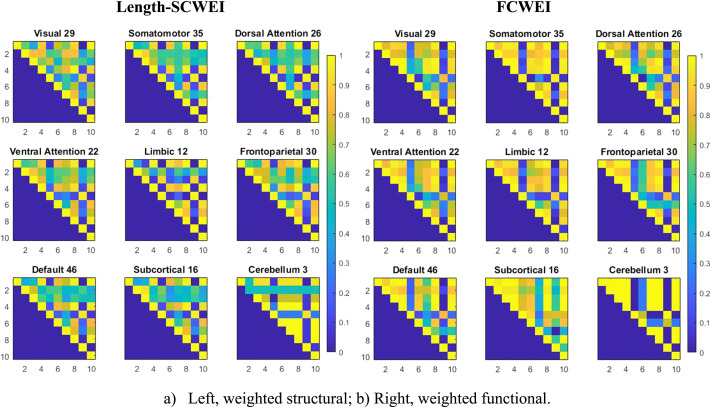
Spearman correlations between nodal properties at the RSN level for three connectome models. (A) Left, weighted structural. (B) Right, weighted structural. Spearman’s correlations computed average across subjects. Since the matrices are symmetric, only the upper triangular part is shown for visual simplicity. The nine matrices on the left, middle, and right represent the nine RSNs of the Length-SCWEI and FCWEI, respectively. The number next to each RSN indicates the number of nodes from the whole network that belongs to the RSN. Nodal property nomenclature; 1 = degree, 2 = betweenness centrality, 3 = closeness centrality, 4 = eigenvector centrality, 5 = clustering coefficient, 6 = participation coefficient, 7 = within-module degree z-score, 8 = PageRank centrality, 9 = average shortest path, and 10 = subgraph.

Since the 10 nodal properties are the building blocks of the graph space, the first question we asked was the following: To what extent are these nodal properties correlated? To answer this question, we computed the Spearman’s rank correlation coefficient (*ρ*) between every pair of nodal properties, resulting in 10 *×* 10 correlation matrices, one for each model (see Supporting Information Figure S1). The same correlations were also computed at the RSN level. Figures 2A and 2B illustrate the Spearman correlation patterns for each RSN of the following connectome models: weighted structural, weighted functional, and binary functional weighted by the structure. The Supporting Information Figures S2A and S2B show results for the three other models. These results chart an atlas of nodal properties where we can see which pairs carry redundant information, for the different RSNs and connectome models. The results show, respectively, positive and negative Spearman’s *ρ* values (in yellow and blue in Figure 2). As expected, for almost all models and RSNs, the clustering coefficient and average shortest path length correlate negatively with all the other nodal properties and have a strong positive correlation. The results at the RSN level show a general pattern of strong positive correlation between the clustering coefficient and average shortest path length; strong positive correlations between degree, betweenness centrality, closeness centrality, eigenvector centrality, within-module degree *z*-score, PageRank centrality, and subgraph; and moderately positive correlations between clustering coefficient and participation coefficient, for most of the RSNs. We also noted differences between RSNs within the same connectome models, such as the correlations of betweenness centrality and other invariants that change depending on the RSNs.

### ML Classification of Brain Regions in Multidimensional Graph Spaces

**Figure 3.**
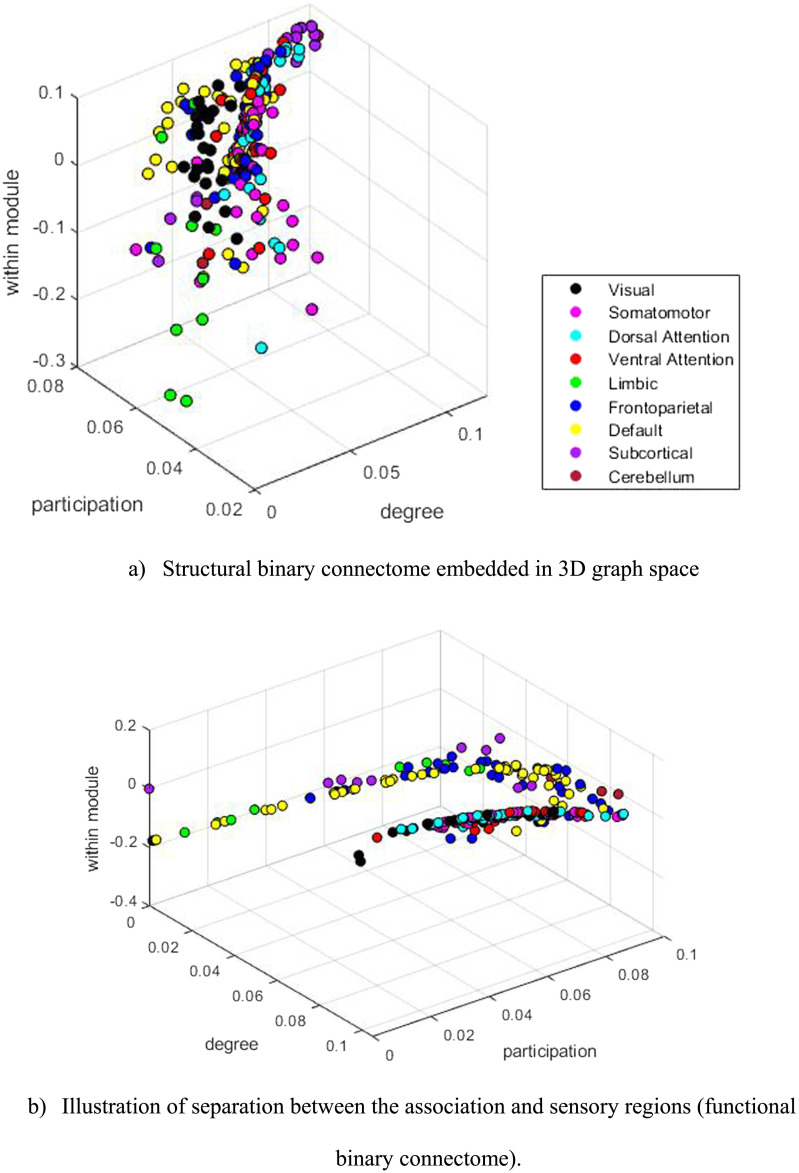
Examples of 3D graph spaces. (A) Structural binary connectome embedded in 3D graph space. (B) Illustration of separation between the association and sensory regions (functional binary connectome). Each dot represents a brain region colored according to the RSN it belongs to. The three axes of the 3D graph space correspond to the degree centrality, participation coefficient, and within-module degree *z*-score nodal properties. The axis (nodal properties) in (A) and (B) were chosen randomly to be properties with different levels of correlations; others could be used. Panel (A) was the first graph space to be computed, and panel (B) exhibits two bands that started the idea of applying ML to graph spaces.

Next, we constructed multidimensional graph spaces for each model. Two examples are given in Figure 3, where we embed group-representative structural and functional connectomes into a 3D graph space built from degree centrality, participation coefficient, and within-module degree *z*-score invariants. From a visual inspection, brain regions tend to form distinct clusters in this space. For example, in Figure 3B, we identified two clusters of brain regions. The first cluster mostly includes brain regions from the subcortical and cerebellum areas and higher-order brain networks, including the limbic, frontoparietal, and default mode RSNs (sub-RSNs 1). The second cluster contains somatosensory areas belonging to the visual, somatomotor, dorsal, and ventral attention RSNs (sub-RSNs 2). To gauge the distance separating these bands, we selected two sets of data points (see Supporting Information Figure S3), computed the Euclidean distance for each pair, and then made an average across those values. This resulted in a 0.0969 estimated distance between the bands. We divided the RSNs into two categories for subsequent ML classification of brain regions in sub-RSNs 1 (limbic, frontoparietal, default mode, subcortical, and cerebellum RSNs) and sub-RSNs 2 (visual, somatomotor, dorsal attention, and ventral attention RSNs) regions. Qualitatively, sub-RSNs 1 regions appear to have greater within-network connectivity, and sub-RSNs 2 regions have greater between-network connectivity (see Figure 3B).

**Figure 4.**
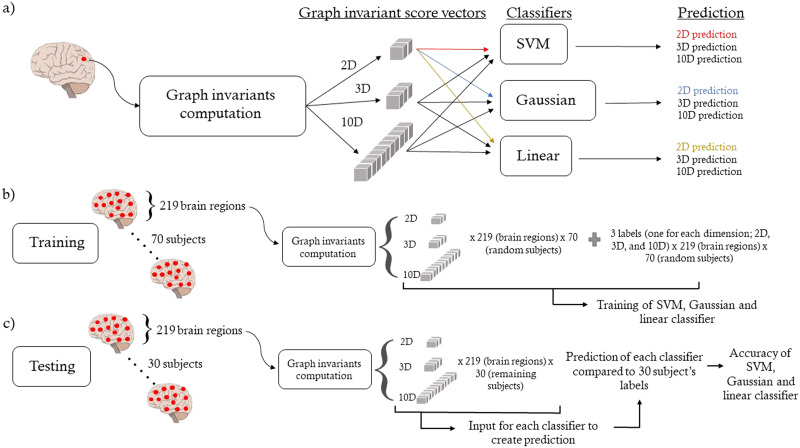
Schematic of ML analysis. (A) Overview of ML pipeline for one brain region. After computing all graph nodal properties, 2, 3, and all 10 nodal properties of the brain region are used for each classifier resulting in a 2D, 3D, and 10D prediction. (B) Training pipeline. Each classifier was trained with 2, 3, and 10 graph nodal properties for all 219 brain regions of 70 randomly selected subjects. This resulted in 219 × 70 = 15,330 brain regions and labels for each dimension (2D, 3D, 10D). (C) Testing pipeline. 219 × 30 = 6,570 brain regions from the remaining 30 subjects were used as input for the classifiers to produce 6,570 predictions. These predictions were compared with the labels to compute the classifier’s accuracy. This was done for all dimensions (2D, 3D, 10D).

We used supervised ML to test whether we could automatically classify brain regions into the categories. We implemented the following classifiers: a binary support vector machine (SVM), a binary Gaussian kernel, and a binary linear classifier. The fitcsvm, fitckernel, and fitclinear MATLAB functions (MathWorks Inc., 2022a, 2022b, 2022c) were used to create each classifier. For each model, all hyperparameters were optimized automatically by MATLAB, setting the OptimizeHyperparameters value to auto. In all cases, classifiers were trained with all the brain regions from 70 randomly selected subjects and then tested on all the brain regions of the remaining 30 subjects. Therefore, our trained models were tested on unseen data. In our case, training an algorithm on 70 randomly selected subjects means that all 70 subjects’ brain regions’ scores are used as input. In other words, each algorithm used a (70 × 219 = 15,330) × *n* matrix as input. The matrix’s rows correspond to the brain regions of the 70 randomly selected subjects, and the *n* columns represent the different nodal property scores (*n* = 10 in a 10D graph space). The ML task is the following: Given multiple brain regions’ graph space coordinates (rows of the input matrix) and categories’ labels (sub-RSNs 1 vs. sub-RSNs 2 regions), can the algorithm predict to which category the unseen brain areas belong? The unseen data used for testing is of shape (30 × 219 = 6,570) × *n*. Each algorithm was trained and tested on different graph space dimensions (2D, 3D, 10D) to see if the prediction increased with the dimensionality, based on the idea that a higher dimensional graph space would yield more brain network information. An overview of our ML approach can be seen in Figure 4.

We trained the models in 2D (degree and clustering), 3D (degree, clustering, and participation), and 10D spaces. The nodal property’s choice for the 2D and 3D spaces was based on the correlation patterns (Supporting Information Figure S1): two invariants with usually opposite correlations across all models (degree and clustering) and the same two invariant plus an invariant showing lower correlations (participation), respectively. The accuracy of each classifier can be seen in Table 1, which is defined as the number of correctly classified brain regions divided by the total number of areas. Indirectly, these accuracy values indicate how suitable the graph space can be when trying to cluster brain areas. In our case, it directly measures how ML approaches can separate brain regions in abstract multidimensional graph spaces. As seen in Table 1, the highest test and train accuracy (86%) was achieved using a binary Gaussian classifier on the FCWEI connectome model in a 10D metric space. The highest accuracies were achieved using all the 10 nodal properties and ranged from 80% to 86%.

**Table 1.**
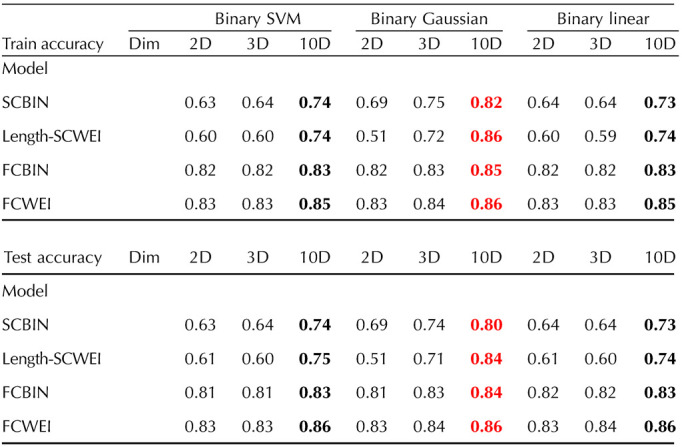
ML classification between sensory and association network nodes

**Bold** numbers are the highest of the three dimensions for each algorithm and model. The red numbers represent the highest accuracies of all the corresponding rows. Dim, dimension.

**Figure 5.**
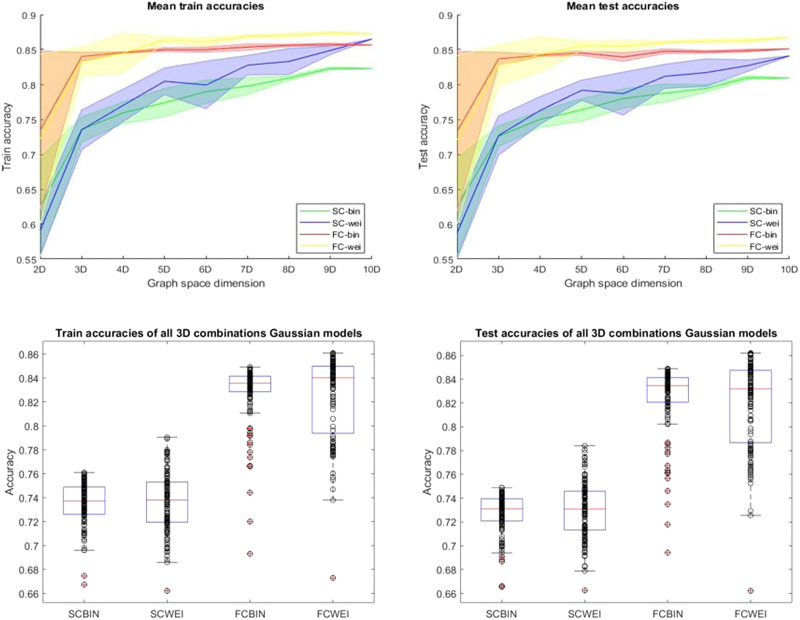
ML train and test accuracies for all connectome models in graph spaces ranging from 2 to 10 dimensions and 3D Gaussian models on all combinations of 10 nodal properties. The first row represents the train (left) and test (right) accuracy curves of binary Gaussian classifiers with different nodal properties (features, which represent the graph space dimension) as input. Except for the 10D graph space, the classifiers were trained with five combinations of randomly selected nodal properties, for each dimension. Each curve represents the mean and the standard deviation of different connectome models. The bottom row represents the train (left) and test (right) accuracies of Gaussian classifiers with all possible combinations of three nodal properties as input. Each dot represents a single classifier’s accuracy (train or test) with three specific nodal properties. These results express how wide the accuracy range of a classifier can be based on a different combination of nodal features. The top left represents the mean of the train accuracies, and the top right represents the mean of the test accuracies.

To better understand the increase in accuracy from a 2D to a 10D graph space, we trained Gaussian classifiers on graph spaces of dimension 2D, up to 10D, in 1D increments. We used Gaussian classifiers since they were the ones illustrating the highest results. For graph spaces of dimensions 2 to 9, the classifiers were trained with five combinations of randomly selected nodal properties, and all 10 features were used for the 10D classifiers. Figure 5 shows the train and test accuracies of all Gaussian classifiers versus graph space dimensions. We also report the best and worst nodal feature combinations for each dimension, and connectome model, in the Supporting Information Figure S6 where the betweenness, participation, and within module consistently emerged as the less-effective combination, contrasting with PageRank and eigenvector features that frequently feature in the most optimal combinations.

Taking into account that for dimensions 2 to 9, not all combinations of nodal features were used for training, and testing, of the classifiers; these results yield more details regarding the amount of information gained with the addition of new nodal features for our classification task (measured as ML accuracy). Supporting Information Table S4 shows which nodal features were used in each Gaussian classifier. We also computed the receiver operating characteristic (ROC) curve and reported the area under the curve (AUC) for each classifier in Supporting Information Figure S6. Looking at the curves in Figure 5, the highest increase in accuracy between two graph space dimensions is from 2D to 3D before exhibiting a plateau in accuracy. Hence, we trained Gaussian classifiers on all combinations of three nodal features to explore all possible 3D graph spaces since three features seem to be enough to reach a high level of accuracy. This resulted in 120 for each connectome model. The train and test accuracies of each classifier can be seen in Figure 5, and the three best, and worst, nodal combinations for each connectome model, as well as the correlation matrices of nodal features, are illustrated in Supporting Information Figure S7.

As additional analysis aimed at comprehending the impact of feature addition in graph spaces, we computed ML accuracy curves for all possible combinations of nodal features within each dimension for the structural binary network. This process generated 45 classifiers in 2D, 120 in 3D, 210 in 4D, 252 in 5D, 210 in 6D, 120 in 7D, 45 in 8D, 10 in 9D, and 1 in 10D, as determined by the possible combinations. However, due to constraints in computational resources and time, this analysis was limited to the structural connectivity (SC) binary model. The results illustrated in Supporting Information Figure S8 offer an insight on the influence of nodal feature combinations on ML accuracy. This analysis complements the original accuracy curves and the exploration of 3D nodal combinations since it combines both analyses and presents a clearer understanding of Table 1. Consistent with our previous ML findings, the most substantial increase in accuracy still occurs upon the addition of a third feature. On a more novel point, in this analysis, there appears to be less of a plateau, and the highest overall accuracies were achieved with nine features. This suggests that a decrease in accuracy may occur depending on the nature of the feature introduced.

To explore how the different nodal features were related, we projected 10D graph spaces into lower dimensional (2D and 3D) latent spaces. We first applied a principal component analysis (PCA) on the 10D graph spaces, which projects the data into a new space where the axes are the principal components that explain most of the data’s variability. The axes of these latent spaces are composed of a weighted combination of the original 10 nodal features. Supporting Information Figures S10A and S10B illustrate the 2D and 3D PCA latent spaces. Here, the 10 vectors represent a nodal feature, and the direction and length of the vector indicate how much the associated nodal feature contributes to the principal component axes. This gives insights into how the 10 nodal features are related to one another when explaining the variability in our data. From the results, the nodal features seem to form three to four groups, indicating that only three to four features could explain a large portion of our data’s variation. This is in accordance with our ML results (Figure 5 and Supporting Information Figures S5, S8, and S9), where the highest gain in accuracy was after adding a third or fourth graph feature.

In addition to the PCA analysis, we also applied a Laplacian embedding on our data since PCA has limitations when handling complex nonlinear data, as previously reported in the literature (van der Maaten et al., 2009). If data points are close in a high-dimensional space, the Laplacian embedding retains this information when performing a latent projection. From the 10D graph space data, we created a network using a k-nearest neighbors algorithm, and the first eigenvectors of the Laplacian matrix of this network are the axes of the latent space. Each brain region can then be associated with a position in the latent space. The 2D and 3D Laplacian embedding results can be seen in Supporting Information Figure S10C, where each dot represents a brain region. These results illustrate the complex nonlinear relationship between the nodal features. PCA and the Laplacian embedding were applied on the average networks for each connectome model. These results provide illustrations of graph spaces into lower dimensional latent spaces.

### Spatial Comparison of Brain Regions of Different Subjects in a Multidimensional Graph Space Using Euclidean Distance

The final analysis of this study was to investigate the distance between brain regions in the multidimensional graph spaces. Each brain region has a set of coordinates (its graph nodal property scores), and we compute the distance between points using the Euclidean distance *d*:

$$d\left(x_{i},y_{i}\right)=\sqrt{\sum_{n=1}^{10}{\left(x_{i,n}-y_{i,n}\right)}^{2}}$$ (1)

where *n* is the invariant, *x* and *y* are two distinct subjects, and *i* is a specific brain region. Hence, *x*_*i*_ and *y*_*i*_ are vectors with 10 values each, and *d*(*x*_*i*_, *y*_*i*_) represents the distance between the two brain regions, *x*_*i*_ and *y*_*i*_, embedded in the 10D graph space. Computing the Euclidean distance for each brain region between two different connectomes gives a vector of 219 values, each representing the Euclidean distance between the same brain region of different subjects. The distances between every pair of subjects were computed, allowing the comparison between different subjects. All these distances formed a 1002 = 4,950 *×* 219 matrix for each connectome model. Averaging across all comparisons resulted in a total distance vector of 219 values for each model. For each model, these vectors represent the average intersubject distance of each brain region. The results are shown in the Supporting Information Figure S11. Furthermore, to mitigate the effect of potential outliers, we computed the ratio between the mean and the standard deviation of its pairwise intersubject distances for each brain region. These results can be seen in the Supporting Information Figure S11.

For the structural models, regions of the default mode network exhibit the highest intersubject distances in the binary connectomes, and temporal regions in the weighted connectomes. The pattern is less clear in the functional models; nevertheless, the frontoparietal and temporal areas always yield relatively high intersubject distances, suggesting that these regions carry the largest proportion of interindividual variability in nodal topological embedding within multidimensional graph spaces. The highest intersubject distances concern the functional and mixed models (FCBIN and FCWEI), while the lowest distances concern the structural models (SCBIN and Length-SCWEI). This result suggests that the functional and structure–function mixed information differentiates subjects better than the structural information alone.

To estimate the difference in graph space distance in the original data versus random models, we compared the 10D intersubject variabilities from our data with the same dimensional intersubject variability from two randomized models created from the Brain Connectivity Toolbox (Brain Connectivity Toolbox, 2022). The first model, Random 1, is a randomization of the edges of a network but preserves the degree distribution. The randomization process occurs by rewiring each edge a certain number of times, five in our study, and was achieved using the “randmio_und()” function, which is based on Maslov and Sneppen’s approach (Maslov & Sneppen, 2002). The second model, Random 2, preserves the strength distribution of a network in addition to the degree distribution. The edges were also rewired five times via the “null_model_und_sign()” function for this method. These models were used since they are standard in the field and because they preserve essential features of our networks. A comparison with a completely random network would yield weaker information regarding the statistical significance of the results. However, comparing random models retaining specific network features, such as degree distributions, results in a more compelling outcome. The statistical significance in this case is not a result of global network differences but, instead, of more complex structures specific to brain networks. One thousand random networks were created for each model, resulting in 2,000 random networks.

**Figure 6.**
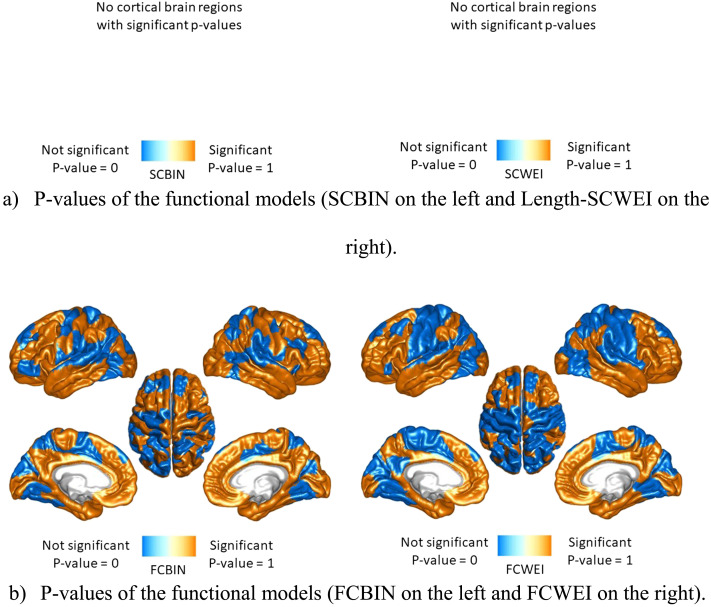
Illustration of the significant versus nonsignificant single region *p* values, after FDR correction, from random model 2. (A) *p* values of the functional models (SCBIN on the left and Length-SCWEI on the right). (B) *p* values of the functional models (FCBIN on the left and FCWEI on the right).

For both random models, a null distribution was generated for each brain region by computing the single nodal distance between every pair of random networks, resulting in a total of 499,500 distances for both Random 1 and Random 2 models. Consequently, for each of the 219 brain regions, an average single nodal distance was derived from the comparison across all 100 subjects (averaging 4,950 distances) and 499,500 distances from the 1,000 random networks. For a single brain region, Supporting Information Figure S14 depicts the null distributions of both random models and the single brain region distance in the form of histograms, along with the average intersubject nodal distance of the corresponding brain region from our HCP data, indicated by the green vertical line. Distributions have been rescaled for the total elements to estimate the probability density function. The number of null distances surpassing the intersubject single nodal distances from our data for each brain region was calculated. This represents the percentage of the null histogram with values higher than the green line in Supporting Information Figure S14. Following the statistical methodology employed in a previous study (Griffa et al., 2023), these percentage values were interpreted as *p* values and corrected for False Discovery Rate (FDR), resulting in a binarization into 0 (nonsignificant; shown in blue) and 1 (significant; shown in orange) for improved visualization. Consequently, this yielded a significant versus nonsignificant outcome for every brain region, with Figure 6 presenting the results for Random 2 and Supporting Information Figure S14 for Random 1. As a second approach, a *z*-scoring to the corresponding null distributions was applied to the intersubject nodal distances. For each brain region, the mean of the associated null distribution was subtracted from the intersubject nodal distance. The result was then divided by the standard deviation of the same null distribution. The resulting *z*-scores (one per brain region) were thresholded at 1.96 to provide a screening of the distances, and the results can be seen in the Supporting Information Figure S15 and S16. This more qualitative method was also employed in a previous study (Griffa et al., 2023). While the results show mostly no significant single brain region distances regarding structural networks, they indicate that several brain regions statistically differ from the null model, particularly for association cortex nodes, in the functional connectivity (FC) data, which correspond to areas previously shown to have “long-range” FC properties (Sepulcre et al., 2010).

After investigating interindividual distances between homologous brain regions, we computed the average interindividual distance of all 219 brain regions to get a single global distance measure between connectome pairs. This resulted in 4,950 global distances, one between every pair of subjects, which were then averaged. This value was computed at multiple network densities to determine how fast these distances would increase or decrease as a function of density for each model. The results for each independent model and all models together can be seen in the Supporting Information Figures S17 and S18. Here, the density of our connectomes is just the amount of strongest connections kept concerning all connections. These results are to be taken as proof that a single global distance between different connectomes can be computed. In our results, the implication of these global distances and curves will not be discussed here since some graph invariants can return aberrant values at low densities.

## DISCUSSION

In this work, we explored multidimensional graph spaces to study connectomes, and our method could be conceptualized as a generalization of previous 2D and 3D graph nodal property approaches (Joyce et al., 2010; Zuo et al., 2012). Our results provide the following new information: (a) graph nodal properties are correlated or anticorrelated at different levels (i.e., there exists specific information between invariants); (b) ML algorithms can separate information in multidimensional graph spaces, especially in high-dimensional spaces; (c) quantification of information increase, measured as classification accuracy, based on several nodal features; (d) visualization of high-dimensional brain network data in different latent spaces; (e) embedding brain regions in a Euclidean graph space provides a mathematical definition of distance between brain regions at multiple scales; and (f) multidimensional graph spaces offer a new way to compare two, or more, connectomes from different subjects. We believe our graph spaces methodology could also be helpful in computational neurosciences.

The correlation patterns showed differences in graph invariants. The changes in patterns at the whole brain network level, as illustrated in Supporting Information Figure S1, can be elucidated by looking at the differences between connectome models (SCBIN, Length-SCWEI, FCBIN, FCWEI). Either the structure of the networks is different (the binary structural and functional networks of the brain are known to be different (Fornito et al., 2016; Sporns, 2016)) or the types of weights (physical vs. topological distances) are different. Both can influence the computation of the invariants. The divergent correlation patterns between RSNs (Figure 2) underline the importance of nodal graph measurements. Each RSN is known to be involved in different cognitive and biological functions (Damoiseaux et al., 2006; Seitzman et al., 2019; van den Heuvel & Sporns, 2013). This suggests that a brain region’s importance must be measured considering its subnetwork environment. In other words, each RSN should be studied with care regarding the choice of nodal invariants, and the relevance of a brain region in its RSN may not always be compared with areas from other RSNs using the same invariants.

The graph space has proven to be well-suited for ML applications. Our work achieved 86% accuracy when classifying brain regions between sub-RSNs1 and sub-RSNs2 areas. Our methodology offers an approach to studying brain areas because they can naturally form clusters in graph spaces. This brings a unique network classification of these areas instead of a biological one. It supports the idea that the graph space allows network nodes to isolate, cluster, or spread themselves spontaneously without any prior hypothesis. All three different ML regimes tested achieved decent results (Table 1), and almost all the best results came from the Gaussian classifier. This indicates that nonlinear-shaped algorithms are better suited for brain network classification in graph spaces. Another noteworthy aspect is that our ML approach not only was computed on whole brain connectomes but can also be used with subnetworks. For example, if a disease is known to target specific brain areas, only networks involving these areas can be studied via graph spaces. Thus, these spaces can be used with networks representing a different level of brain complexity (whole brain, subnetworks, fewer or more nodes for the same scale, etc.), showing the adaptability and flexibility of the proposed tool to match researchers’ needs. Our accuracy results consistently support the notion of increased information with higher dimensions, although the rate of accuracy increase is not linear, as evidenced in Figure 5 and Supporting Information Figures S8 and S9. According to our findings, the most significant improvement in accuracy, and therefore, information gain, occurs when transitioning from a 2D to a 3D graph space. For functional models, employing more than three nodal features leads to marginal increases in accuracy, as indicated by the plateau observed in both FCBIN and FCWEI accuracy curves. A similar trend is observed in structural models, where the bulk of accuracy enhancement occurs with three nodal properties, followed by a more gradual increase with additional features. These results suggest that utilizing three nodal properties is generally sufficient for achieving reasonably accurate classifications. Upon analyzing all 3D graph spaces (refer to Figure 5 and Supporting Information Figure S7), we identify the three best and worst feature combinations for our ML classification task. Notably, functional models exhibit slightly greater variability, particularly the FCWEI model, implying that more caution should be exercised in selecting nodal features when constructing a functional connectome graph space.

Our current ML approach has some limitations, the biggest being that K-fold cross-validation should be implemented to reach better accuracy since our results come from hold-out cross-validation. The number of subjects used (100) could also be increased, and more sophisticated ML methods could be used, such as neural networks. Nevertheless, we show preliminary evidence that more extensive information, understanding, and better classification could come from these networks when at least three dimensions are in place.

Finally, we proposed a new distance measure in the graph space to compare single brain regions and whole brain comparison (Figure 6 and Supporting Information Figures S11, S18, and S19). It is known that connectomes of different subjects express differences between them (Varoquaux & Craddock, 2013; Van De Ville et al., 2021); however, graph spaces offer a different angle to explore discrepancies between connectomes, via multiple network algorithms. The distance across subjects’ results expresses how topologically far brain regions are from each other, from a network perspective. More specifically, if the distance between the same pair of regions from different subjects is high, this region is not located at the same position for both subjects in the graph space. Hence, there is a network difference between both subjects. Based on our results (Figure 6 and Supporting Information Figures S10, S15, and S16), structural and functional distances may be used to understand the brain’s distance patterns further. The structural distances yielded high values in the default network areas and low values in frontoparietal and visual networks. The high values in the default network could come from elements comprising this RSN that are spread across the brain (Seitzman et al., 2019; Shulman et al., 1997; van den Heuvel & Hulshoff Pol, 2010) and, thus, could differ structurally (in length or strength) between different people. The functional distances appeared to be highest in the frontoparietal and temporal areas and lowest in the somatomotor and dorsal networks. The frontoparietal regions are known to be involved in complex cognitive activities (Marek & Dosenbach, 2018); thus, it is reasonable that these regions are subject-specific and express high distances from each other in fMRI data.

Whole distance results are not straightforward to interpret since we lose some information, having only one distance value. Nevertheless, a trend is that networks express lower distances at high density. Also, functional networks exhibit higher distances than structural models, expressing that functional brain information is more subject-specific than structural data. In other words, the physical distance used in the structural connectomes is more similar across subjects than the topological functional distance. This could come from the fact that the global, underlying brain structure is relatively similar across humans, even though differences can be found at multiple connectivity levels (Sporns et al., 2005).

We believe that multidimensional graph spaces are tools that could permit researchers to discover brain patterns. Closest neighbor, topological data analysis via persistent homology and Betti numbers, and state-of-the-art ML techniques (Bagrow & Bollt, 2019; Berlingerio et al., 2012; Grover & Leskovec, 2016; Mheich et al., 2020; Narayanan et al., 2017; Takahashi et al., 2012) are examples of future research areas. Regarding the ML approaches, analyses of clinical data could help identify cluster-specific brain regions involved in neurological diseases nonincisively. More sophisticated ML methods, such as neural networks, should also be investigated to see if they could better locate brain data patterns embedded in high-dimensional graph spaces. Also, ML analyses of subnetworks can offer a new perspective on identifying network differences and patterns extractable from and across them. Studies have already investigated connectome comparisons between different species (Faskowitz et al., 2023; Mheich et al., 2020), but this could also be a future research area regarding graph spaces. Another relevant question for future work is whether nodal properties are redundant when gaining new information. We argue here that high-dimensional spaces are well suited for studying connectomes, but does each new dimension (each nodal property) give us more information than we already had in lower dimensional spaces? Two types of answers can be considered in this context: (a) Some nodal properties do not improve the knowledge of the connectome in a graph space sense, leading to a debate over which nodal properties convey the most information, or (b) any new nodal measure computed can either increase the amount of global information or equal it, but never decrease it. Our preliminary results suggest that the increase in performance, as features are added, is not linear since most of the accuracy increase happens after the third graph space dimension.

Another question regarding ML in graph spaces is how it compares with state-of-the-art graph ML or other comparison methods (Mheich et al., 2020). Examples include node2vec (Grover & Leskovec, 2016), which uses nodal values of a graph to learn a vector representation of nodes via random walks and word embedding tools; graph2vec (Narayanan et al., 2017), which relies on representation learning to perform whole graph embedding; NetSmile (Berlingerio et al., 2012), which uses seven predefined graph features and Singular Value Decomposition to output a similarity score between whole networks; network portrait divergence (Bagrow & Bollt, 2019), which uses network portraits and information theory to compare networks; or graph spectral entropy (Takahashi et al., 2012) that uses the eigenvalues of the adjacency matrix to compute an entropy graph score, which can be used to compare graphs. All these methods have their advantages and can be used for different purposes. However, it is nontrivial to compare our approach with these methods as global distances resulting from graph spaces are hard to interpret and have not been tested with subject classification. Still, this topic should be revisited in detail in future studies.

In conclusion, we explore graph spaces in the context of neuroscience. However, as mentioned previously, it can be used with any network; therefore, it is not just a fruitful tool in computational neuroscience but it could lead to novel findings in any other field involving the use of networks.

## METHODS

### Dataset and Preprocessing

This study used a Human Connectome Project dataset of structural MRI and fMRI data from 100 unrelated subjects (Van Essen et al., 2013). All diffusion MRI data were processed following the main MRtrix guidelines (Tournier et al., 2019) with two modifications: (a) The track seeding was set to 20 million tracts, and (b) the algorithm “spherical deconvolution informed filtering of tractograms” 2.0 (Smith et al., 2015) was used.

The fMRI data included four sessions of resting state acquired for each subject (two per day with opposed phase encoding direction), each composed of time series of 1,200 acquired volumes (TR = 0.72 s between volumes) that were preprocessed as in a previous study (Van De Ville et al., 2021). Briefly, preprocessing steps included linear and quadratic detrending, removal of motion regressors and their first derivatives, removal of white matter, cerebrospinal fluid average signals and their first derivatives, and a bandpass filter in the range of 0.01–0.15 Hz.

### Connectomes

The 200 cortical regions of the Schaefer parcellation (Schaefer et al., 2018), plus 19 subcortical and cerebellum regions (as in Griffa et al., 2022), were used to create the connectomes.

The edges of both structural connectomes (SCBIN and Length-SCWEI) represent anatomical connections between the brain regions. Two graphs were created using two metrics as edges: fiber length and the number of streamlines (Griffa et al., 2023). The fiber length matrices represent the distance separating two brain regions in millimeters, and the number of streamline matrices expresses the strength of the connection between different brain areas. A consensus mask across all subjects was computed by binarizing the number of streamline matrices. This resulted in a matrix where the *ij*-th element is 1 if there is a connection between brain regions *i* and *j* across all subjects; otherwise, it is 0. Next, the average of all 100 fiber length matrices was multiplied with the consensus mask, giving an average masked fiber length matrix. This matrix is a structural connectome created using all subjects’ structural information. The density of this average structural connectome was used to obtain single-subject structural connectomes, where individual fiber length matrices were thresholded to keep only more robust connections and match the average connectome density. The connections retained are the edges associated with the smallest weights (here, weights represent distances). Therefore, these edges represent physically proximate brain regions. The Length-SCWEI model considers these single-subject fiber length matrices, while the SCBIN model considers the same matrices but binarized. The reason for using the fiber length as weights for the structural model was motivated since we wanted to use both the number of streamlines and fiber length information.

For the functional connectomes (FCBIN and FCWEI), the edges do not represent a physical connection but a statistical correlation between fMRI signals of different brain areas. The pairwise correlations between regional average time series were computed and considered as edges of a functional connectome. The information these matrices convey does not refer to physical links between nodes but rather how similar their activity pattern is. Conversely, if the activity of two brain regions is desynchronized, their correlation will be low. The absolute value of each element was computed, and the density of the average masked fiber length matrix was again used as a threshold to keep only the most robust connections. Furthermore, the FCWEI model is the single-subject matrix with the original correlation numbers, while the FCBIN model is the same matrix but thresholded to have a binary matrix.

A remark should be made on the fact that thresholding of data, specifically functional data, can wrongly generate graph properties (Cantwell et al., 2020; van den Heuvel et al., 2017). This can also result in aberrant nodal properties if the networks are composed of multiple components. To address this issue, we also computed the ML accuracies, mean single region distances, and the *z*-scored single brain distances on unthresholded structural and functional networks. The results are in the Supporting Information Figures S9 and S13. The ML findings demonstrate a distinct pattern compared with thresholded matrices (Figure 5), showing a smaller increase in accuracy between 2D and 3D nodal features and a less prominent plateau effect with three or more nodal features. These results suggest that for unthresholded FC and SC matrices, the precision accuracy primarily improves between two and five nodal features, with a slower rate of accuracy improvement observed beyond five nodal features. Structural average intersubject distances illustrate a similar pattern than our previous results (Supporting Information Figure S11), but motor regions tend to have higher values for functional distances. *Z*-score results do not yield any clear patterns. These analyses illustrate how graph spaces can be used for both threshold and unthreshold FC and SC matrices.

### Graph Invariants

Let *G* be a graph with *n* vertices, ∣*V*∣ = *n*. The adjacency matrix *A* of *G* is an *n* × *n* symmetric matrix where each element *a*_*ij*_ is defined as,

$$a_{ij}=\{1\;\text{if}\;\left(v_{i},v_{j}\right)\in E\;0\;\text{otherwise}$$

A graph nodal property is an algorithm that takes as input a graph, *G*, and computes a score, *s*_*i*_, for each vertex belonging to *V*. Each vertex score represents how vital this vertex is in the whole graph. The 10 following nodal properties were used in this study.

***Degree***:

The degree nodal property, *C*_*D*_, of a vertex *i* is defined as the number of neighbors of *i*. *C*_*D*_(*i*) = *deg*(*v*_*i*_).

***Betweenness***:

The betweenness nodal property, *C*_*b*_, of a vertex *i* is the ratio of the shortest paths connecting any pair of vertices, *s* and *t*, that passes through *i* divided by the total number of shortest paths between *s* and *t*. *C*_*b*_(*i*) = ∑_*s*≠*i*≠*t*_ $\frac{\sigma_{ist}}{\sigma_{st}}$ where *σ*_*sit*_ is the number of shortest paths from *s* to *t* that passes through *i*, and *σ*_*st*_ is the total number of shortest paths from *s* to *t*.

***Closeness***:

The closeness, *C*_*c*_, of a vertex is the inverse sum of the length of the shortest paths connecting the vertex to all other vertices in the graph. *C*_*c*_ = $\frac{1}{\sum_{s}\;d\left(i,s\right)}$, where *d*(*i*, *s*) is the distance between vertices.

***Eigenvector***:

The eigenvector nodal property, *C*_*e*_, is based on the idea that if a vertex *v*_*i*_ is linked to many vertices with a high eigenvector score, it will also have a high eigenvector score, and vice versa if it is linked with lower scores. *C*_*e*_(*v*_*i*_) = $\frac{1}{\lambda }\sum_{s=1}^{n}$ *a*_*is*_*v*_*s*_, where *λ* is the highest eigenvalue of the adjacency matrix *A*.

***Clustering***:

The clustering coefficient, *C*_*clu*_, of a vertex *i*, represents how much the vertices connected to *i* are linked together. *C*_*clu*_ = $\frac{L_{i}}{k_{i}\left(k_{i}-1\right)/2}$, where *L*_*i*_ is the number of edges between the neighbors of *i* and *k*_*i*_ is the degree of *i*.

***Participation***:

The participation coefficient, *C*_*p*_, reflects how much a vertex *i* is linked with vertices of its module or vertices from other modules. Modules can be thought of as clusters of vertices that are highly interlinked. *C*_*p*_(*i*) = 1 − $\sum_{m=1}^{M}\;{\left(\frac{d_{i}\left(m\right)}{d_{i}}\right)}^{2}$, where *M* is the set of all the modules, *d*_*i*_ is the degree of vertex *i*, and *d*_*i*_(*m*) is the degree between vertex *i* and all vertices in module *m*.

***Within-module degree z-score***:

The within-module degree *z*-score, *C*_*z*_, of a vertex *i*, is the *z*-score of *i* within its module. It is related to the degree of a vertex but only within its module. *C*_*z*_(*i*) = $\frac{d_{mi}-\left(\text{mean}\left(d_{mi}\right)\right)}{\sigma_{d_{mi}}}$, where *d* is the degree of vertex *σ*_*d*_*mi*__ in its module *m*_*i*_, *mean*(*d*_*mi*_) is the mean degree of *i* within its module *m*_*i*_, and *σ*_*d*_*mi*__ is the standard deviation of the degree of *i* within its module.

***PageRank***:

The PageRank Google’s nodal property, *C*_*pr*_, is a variation of the eigenvector nodal property but follows the same idea of self-reference. The PageRank score of a vertex *i* can be thought of as the amount of time a random walker would spend on *i* concerning the whole graph and with a damping factor specifying the amount of time the random walker will step on a neighborhood vertex *i*. *C*_*pr*_(*i*) = 1 − *d* + *d*$\sum_{s=1}^{n}\;\frac{a_{\text{is}}C_{pr}\left(s\right)}{d\left(s\right)}$, where *d* is the damping factor, usually 0.85, and *d*(*s*) is the degree of vertex *s*.

***Average shortest path***:

The average shortest path nodal property, *C*_*asp*_, is a measure of the efficiency of a vertex in transferring information in the whole network. It can be defined as the reciprocal of nodal efficiency. *C*_*asp*_(*i*) = $\frac{1}{E_{\text{global}}\left(i\right)}$, where *E*_*global*_(*i*) = $\frac{1}{n\left(n-1\right)}\sum_{s}\;\frac{1}{l_{\text{is}}}$ is the global efficiency of vertex *i*, with *l*_*is*_ being the shortest path between vertices *i* and *s*.

***Subgraph***:

The subgraph nodal property, *C*_*s*_, of a vertex *i* is a weighted sum of a closed walk that can be of different lengths, starting and ending at *i*. *C*_*s*_(*i*) = $\sum_{j=1}^{n}\;{\left(x_{j}^{i}\right)}^{2}$*e*^*λ*_*j*_^, where *x*_*j*_ is the *j*-th eigenvector of *A*, *x*^*i*^ is the *i*-th component of *x*_*j*_, and *λ*_*j*_ is the *j*-th eigenvector.

All the analyses and nodal properties were computed on MATLAB using the Brain Connectivity Toolbox (Brain Connectivity Toolbox, 2022), and after computation, all nodal measures were individually normalized (each property vector was normalized to the highest value of the vector by taking the ratio of each value to the maximum value of the vector, resulting in scaling the data between 0 and 1 and potentially reducing the impact of outliers that could have extreme values). The latent space results (Supporting Information Figure S10) were computed via the MATLAB Toolbox for Dimensionality Reduction (van der Maaten, 2024).

## ACKNOWLEDGMENTS

MGP was supported by the CIBM Center for Biomedical Imaging, a Swiss research center of excellence founded and supported by Lausanne University Hospital (CHUV), University of Lausanne (UNIL), Ecole polytechnique fédérale de Lausanne (EPFL), University of Geneva (UNIGE), and Geneva University Hospitals (HUG). EA acknowledges financial support from the SNSF Ambizione project ‘Fingerprinting the brain: network science to extract features of cognition, behaviour and dysfunction’ (grant no. PZ00P2_185716). RL was supported by the Swiss National Centre of Competence in Research - Evolving Language (grant number 51NF40_180888). MGP was supported by the CIBM Center for Biomedical Imaging, a Swiss research center of excellence founded and supported by Lausanne University Hospital (CHUV), University of Lausanne (UNIL), Ecole polytechnique fédérale de Lausanne (EPFL), University of Geneva (UNIGE), and Geneva University Hospitals (HUG). AG acknowledges funding from the Ernest Boninchi Foundation project “BrainCom—Communication dynamics in system-level brain networks: Novel methodology with application to reversible dementia.” MP acknowledges funding from the Health Research Council of New Zealand (HRC), grant #21/622.

## SUPPORTING INFORMATION

Supporting information for this article is available at https://doi.org/10.1162/netn_a_00393.

## AUTHOR CONTRIBUTIONS

Mathieu Mach: Conceptualization; Formal analysis; Investigation; Methodology; Software; Writing – original draft; Writing – review & editing. Enrico Amico: Resources; Writing – review & editing. Raphaël Liégeois: Resources; Writing – review & editing. Maria Giulia Preti: Resources; Writing – review & editing. Alessandra Griffa: Resources; Software; Writing – review & editing. Dimitri Van De Ville: Resources; Writing – review & editing. Mangor Pedersen: Conceptualization; Investigation; Methodology; Supervision; Writing – original draft; Writing – review & editing.

## DATA AVAILABILITY STATEMENT

The data used in this study is open-source and available at https://db.humanconnectome.org.

## Supplementary Material



## TECHNICAL TERMS

Graph: A network composed of nodes connected via edges.
Connectome: A graph representing the brain with nodes and edges representing brain regions and connections between them.
Correlation: A measure of similarity between two variables, signal, or vectors.
Machine learning: Optimization of algorithms to automatically find patterns in data for prediction.
Euclidean distance: A distance measure between two points.
Structural and functional connectome: Different models of connectome where the edges represent either structural (physical) brain connections or functional (brain activity) correlations.
Nodal feature or centrality: A method or algorithm attributing a score to each node reflecting it’s importance in the graph.
PCA: A method reducing high-dimensional data into lower dimension while keeping the variability of the input.
Laplacian embedding: A data dimensional reduction method based on the Laplacian matrix graph of the data.
*p* value: A probability that, under a null hypothesis, the data observed come from a random distribution.
FDR: A method for correcting the number of significant statistical results when many comparisons are performed.
