# Supplementary material for: Connectome embedding in multidimensional graph spaces
Source: Netw Neurosci. 2024 Dec 10;8(4):1129–48. doi: 10.1162/netn_a_00393 (PMC11674405; doi:10.1162/netn_a_00393)
Supplement: Supplementary file 1 [file netn-8-4-1129-s001.pdf]

Connectome embedding in multidimensional graph spaces

Mathieu Mach[1], Enrico Amico[1,2], Raphaël Liégeois[1,2], Maria Giulia Preti[1,2,3],

Alessandra Griffa[1,4], Dimitri Van De Ville[1,2,3], Mangor Pedersen[5]

August 2022

[1] Neuro-X Institute, Ecole Polytechnique Fédérale De Lausanne (EPFL), Geneva, Switzerland

[2] Department of Radiology and Medical Informatics, University of Geneva (UNIGE), Geneva, Switzerland

[3] CIBM Center for Biomedical Imaging, Switzerland

[4] Leenaards Memory Center, Lausanne University Hospital and University of Lausanne, Lausanne, Switzerland

[5] Department of Psychology and Neuroscience, Auckland University of Technology, New Zealand

Spearman correlations between nodal properties at the whole connectome level.

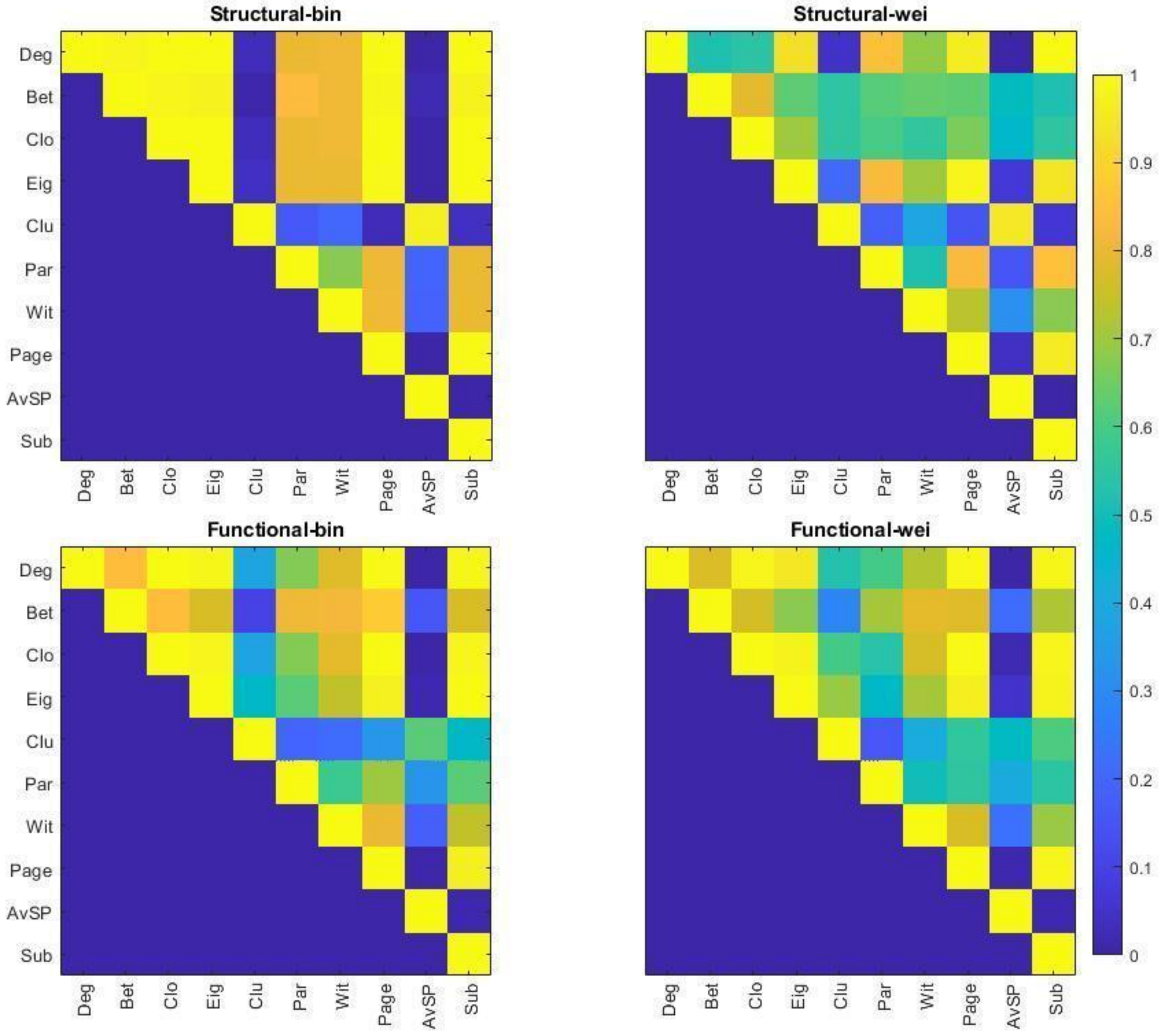

Supplementary Figure 1: Spearman's correlations at whole connectome level, for each model. All correlations were computed on the average level. Since the matrices are symmetric, only the upper triangular part is shown for visual simplicity.

Spearman correlations between nodal properties at RSN level for three different connectome models.

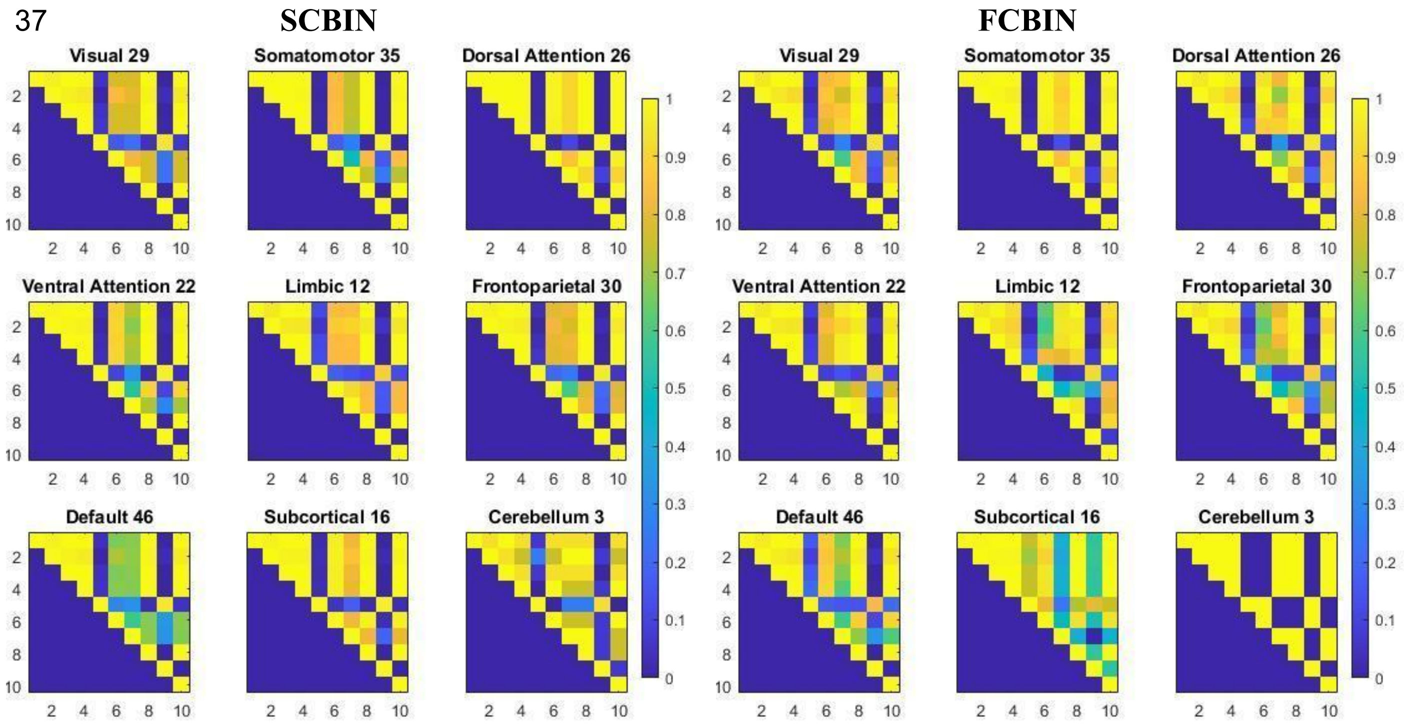

a) Left, binary structural, b) right, binary functional.

Supplementary Figure 2: Spearman's correlations computed on average across subjects. Since the matrices are symmetric, only the upper triangular part is shown for visual simplicity. The nine matrices on the left, middle, and right represent the nine RSN's of the SCBIN, and FCBIN, respectively. The number next to each RSN indicates the number of nodes from the whole network that belongs to the RSN. Nodal properties nomenclature; 1 = degree, 2 = betweenness, 3 = closeness, 4 = eigenvector, 5 = clustering, 6 = participation, = 7 = within-module degree z-score, 8 = PageRank, 9 =average shortest path, and 10 = subgraph.

Quantification estimate of the distance between the two graph space bands.

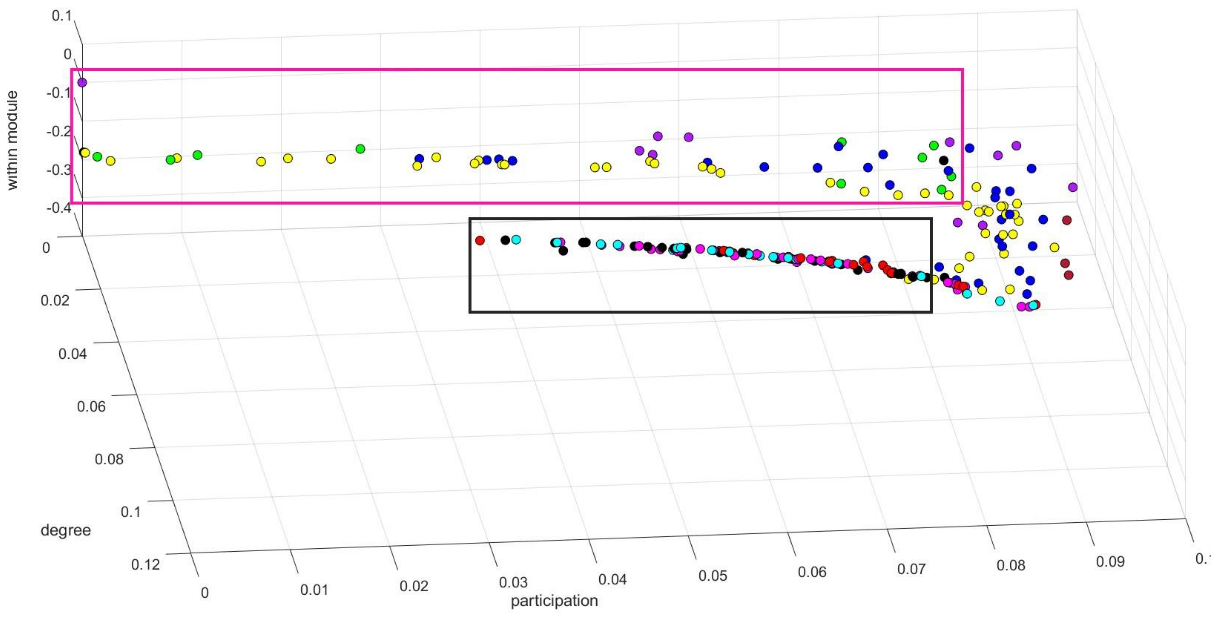

Supplementary Figure 3: Illustration of the 2 sets of data used to compute an estimate of the distance separating the graph space bands.

Nodal feature combinations utilized to compute ML accuracies in Table 1.

Combination \ Dimension	2	3	4	5	6	7	8	9
1	2,9	4,7,8	3,5,7,10	2,3,5,9,10	1,2,6,7,8,9	2,4,5,7,8,9,10	1,3,4,5,6,7,9,10	1,2,3,5,6,7,8,9,10
2	4,3	3,5,6	2,4,6,9	1,4,6,7,8	3,5,6,7,8,9	2,3,4,6,7,8,10	3,4,5,6,7,8,9,10	1,2,4,5,6,7,8,9,10
3	1,8	1,9,10	1,6,7,8	1,4,6,8,9	1,2,3,4,5,8	1,3,4,7,8,9,10	1,3,4,6,7,8,9,10	1,2,3,4,5,6,7,8,9
4	5,6	3,8,10	3,5,9,10	2,3,5,7,10	1,2,4,5,7,9	2,4,5,6,8,9,10	1,2,3,4,5,6,7,9	1,2,3,4,6,7,8,9,10
5	7,1	1,2,9	2,4,5,8	1,2,3,4,8	1,3,4,5,7,10	1,2,3,5,6,7,10	1,2,3,4,6,7,8,10	2,3,4,5,6,7,8,9,10

Supplementary Table 4: Combinations of nodal features used in the Gaussian classifiers of graph spaces of dimensions 2 to 9. Nodal properties nomenclature; 1 = degree, 2 = betweenness, 3 = closeness, 4 = eigenvector, 5 = clustering, 6 = participation, = 7 = within-module degree z-score, 8 = PageRank, 9 =average shortest path, and 10 = subgraph.

Gaussian classifiers train and test ROC curves and AUC for graph space from 2 to 10 dimensions with 5 different combinations of nodal features as input.

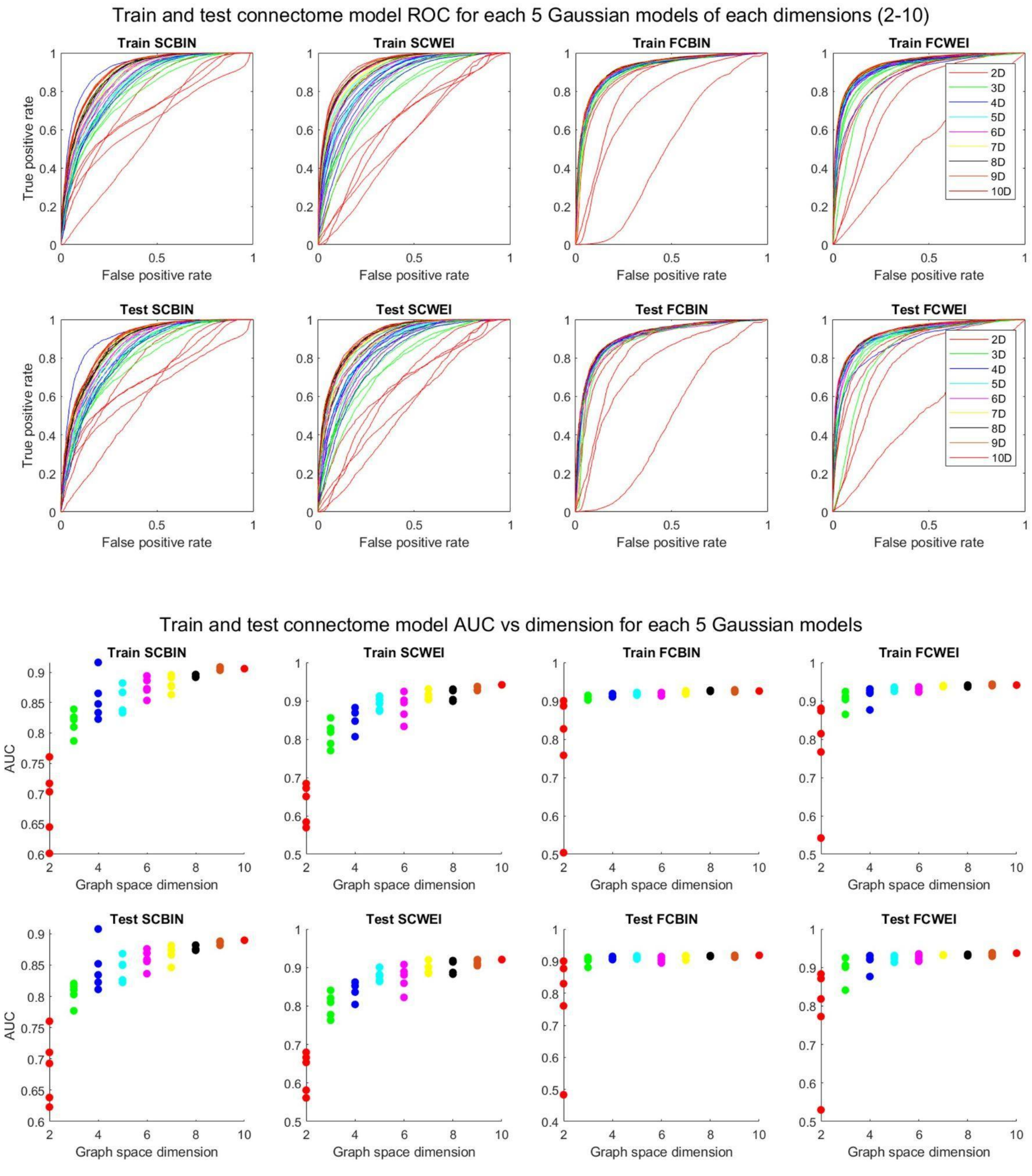

Supplementary Figure 5: First two rows, Gaussian classifiers receiver operating characteristic (ROC) curves in graph spaces ranging from 2 to 10D. Train and test ROC on the first and second row respectively. Third and fourth rows, area under the curve (AUC) where each dot represents the train, or test, area under the curve (AUC) value of a Gaussian classifier in a graph space of a certain dimension and with a specific combination of nodal

properties as input. Train and test ROC on the third and fourth row respectively. For dimensions 2 up to 9, 5 random combinations of nodal properties were used as input.

Best and worst nodal feature combinations for train and test ML accuracies of Figure 5.

**Best**									
		DIMENSION							
Connectome model		2	3	4	5	6	7	8	9
SCBIN		2,9	6,5,3	7,1,8,6	1,6,8,4,7	7,2,8,6,9,1	10,3,2,6,1,5,7	7,5,3,9,4,2,6,1	1,2,3,4,5,6,7,8,9
SCWEI		4,3	3,8,10	9,10,5,3	1,2,4,8,3	5,2,4,3,1,8	3,8,10,4,9,1,7	9,3,8,7,4,1,10,6	1,2,3,4,6,7,8,9,10
FCBIN		1,8	4,8,7	8,2,5,4	1,6,8,4,7	7,2,8,6,9,1	10,3,2,6,1,5,7	7,5,3,9,4,2,6,1	1,2,3,5,6,7,8,9,10
FCWEI		4,3	4,8,7	8,2,5,4	1,6,8,4,7	5,10,7,4,3,1	7,8,10,4,2,6,3	9,3,8,7,4,1,10,6	1,2,3,5,6,7,8,9,10
**Worst**									
		DIMENSION							
Connectome model		2	3	4	5	6	7	8	9
SCBIN		4,3	3,8,10	9,10,5,3	2,9,3,5,10	5,2,4,3,1,8	8,5,2,6,4,10,9	7,1,4,5,6,3,10,9	1,2,3,4,6,7,8,9,10
SCWEI		5,6	9,2,1	7,1,8,6	1,6,8,4,7	7,2,8,6,9,1	7,8,10,4,2,6,3	7,1,4,5,6,3,10,9	1,2,4,5,6,7,8,9,10
FCBIN		2,9	6,5,3	6,4,2,9	2,9,3,5,10	3,8,6,9,5,7	8,5,4,9,10,7,2	7,1,4,5,6,3,10,9	2,3,4,5,6,7,8,9,10
FCWEI		2,9	3,8,10	7,1,8,6	2,9,3,5,10	3,8,6,9,5,7	10,3,2,6,1,5,7	7,3,8,6,4,2,10,1	1,2,3,4,6,7,8,9,10

a) Best (top) and worst (bottom) train nodal combinations for each connectome model and each graph space dimension.

**Best**									
		DIMENSION							
Connectome model		2	3	4	5	6	7	8	9
SCBIN		2,9	1,9,10	7,1,8,6	1,6,8,4,7	7,2,8,6,9,1	10,3,2,6,1,5,7	7,5,3,9,4,2,6,1	1,2,3,4,5,6,7,8,9
SCWEI		4,3	3,8,10	8,2,5,4	1,2,4,8,3	5,2,4,3,1,8	3,8,10,4,9,1,7	9,3,8,7,4,1,10,6	1,2,3,4,6,7,8,9,10
FCBIN		1,8	4,8,7	8,2,5,4	1,6,8,4,7	9,7,1,5,2,4	10,3,2,6,1,5,7	7,3,8,6,4,2,10,1	2,3,4,5,6,7,8,9,10
FCWEI		4,3	4,8,7	8,2,5,4	1,2,4,8,3	5,2,4,3,1,8	3,8,10,4,9,1,7	7,3,8,6,4,2,10,1	2,3,4,5,6,7,8,9,10
**Worst**									
		DIMENSION							
Connectome model		2	3	4	5	6	7	8	9
SCBIN		4,3	3,8,10	9,10,5,3	2,9,3,5,10	5,2,4,3,1,8	8,5,2,6,4,10,9	9,3,87,7,4,1,10,6	2,3,4,5,6,7,8,9,10
SCWEI		5,6	9,2,1	7,1,8,6	1,6,8,4,7	7,2,8,6,9,1	7,8,10,4,2,6,3	7,1,4,5,6,3,10,9	1,2,3,5,6,7,8,9,10
FCBIN		2,9	6,5,3	9,10,5,3	10,5,7,2,3	3,8,6,9,5,7	3,8,10,4,9,1,7	5,3,6,7,4,8,10,9	1,2,3,5,6,7,8,9,10
FCWEI		2,9	3,8,10	7,1,8,6	2,9,3,5,10	7,2,8,6,9,1	10,3,2,6,1,5,7	7,1,4,5,6,3,10,9	1,2,3,5,6,7,8,9,10

b) Best (top) and worst (bottom) test nodal combinations for each connectome model and each graph space dimension.

Supplementary Figure 6: Nodale feature combinations yielding the best, a), and worst, b), accuracy results for each model and each graph space dimensions. For each dimension, the best and worst results come from the 5 randomly selected combinations illustrated in Supplementary Table 4. Nodal properties nomenclature; 1 = degree, 2 = betweenness, 3 = closeness, 4 = eigenvector, 5 = clustering, 6 = participation, = 7 = within-module degree z-score, 8 = PageRank, 9 =average shortest path, and 10 = subgraph.

Best, and worst, nodal feature combinations for train and test ML accuracies of 3D Gaussian models on all possible combinations of 10 nodal properties.

Correlations SCBIN	1	2	3	4	5	6	7	8	9	10
1	1.0000	0.9687	1.0000	0.9948	-0.9406	0.6033	0.6187	0.9994	-1.0000	0.9948
2	0.9687	1.0000	0.9687	0.9472	-0.9893	0.6770	0.6206	0.9754	-0.9687	0.9472
3	1.0000	0.9687	1.0000	0.9948	-0.9406	0.6033	0.6187	0.9994	-1.0000	0.9948
4	0.9948	0.9472	0.9948	1.0000	-0.9144	0.6015	0.5998	0.9915	-0.9948	1.0000
5	-0.9406	-0.9893	-0.9406	-0.9144	1.0000	-0.6790	-0.5975	-0.9489	0.9406	-0.9144
6	0.6033	0.6770	0.6033	0.6015	-0.6790	1.0000	0.3566	0.6102	-0.6033	0.6015
7	0.6187	0.6206	0.6187	0.5998	-0.5975	0.3566	1.0000	0.6237	-0.6187	0.5998
8	0.9994	0.9754	0.9994	0.9915	-0.9489	0.6102	0.6237	1.0000	-0.9994	0.9915
9	-1.0000	-0.9687	-1.0000	-0.9948	0.9406	-0.6033	-0.6187	-0.9994	1.0000	-0.9948
10	0.9948	0.9472	0.9948	1.0000	-0.9144	0.6015	0.5998	0.9915	-0.9948	1.0000

Correlations SCWEI	1	2	3	4	5	6	7	8	9	10
1	1.0000	0.0324	0.0805	0.8558	-0.9028	0.6933	0.3682	0.9189	-1.0000	0.9948
2	0.0324	1.0000	0.5906	0.2516	0.0975	0.2384	0.2743	0.2587	-0.0324	0.0329
3	0.0805	0.5906	1.0000	0.4057	0.1039	0.1970	0.1243	0.3220	-0.0805	0.1043
4	0.8558	0.2516	0.4057	1.0000	-0.5904	0.6497	0.3988	0.9677	-0.8558	0.8829
5	-0.9028	0.0975	0.1039	-0.5904	1.0000	-0.6464	-0.2326	-0.7003	0.9028	-0.8737
6	0.6933	0.2384	0.1970	0.6497	-0.6464	1.0000	0.0321	0.6626	-0.6933	0.7020
7	0.3682	0.2743	0.1243	0.3988	-0.2326	0.0321	1.0000	0.4668	-0.3682	0.3591
8	0.9189	0.2587	0.3220	0.9677	-0.7003	0.6626	0.4668	1.0000	-0.9189	0.9271
9	-1.0000	-0.0324	-0.0805	-0.8558	0.9028	-0.6933	-0.3682	-0.9189	1.0000	-0.9948
10	0.9948	0.0329	0.1043	0.8829	-0.8737	0.7020	0.3591	0.9271	-0.9948	1.0000

a) Mean features correlation of SCBIN (top), and length-SCWEI (bottom).

Correlations FCBIN	1	2	3	4	5	6	7	8	9	10
1	1.0000	0.6981	0.9995	0.9727	-0.1727	0.3723	0.5906	0.9918	-0.8923	0.9727
2	0.6981	1.0000	0.7036	0.5674	-0.7060	0.6413	0.6538	0.7660	-0.5941	0.5674
3	0.9995	0.7036	1.0000	0.9704	-0.1807	0.3758	0.5981	0.9929	-0.8922	0.9704
4	0.9727	0.5674	0.9704	1.0000	-0.0124	0.2844	0.5068	0.9419	-0.8641	1.0000
5	-0.1727	-0.7060	-0.1807	-0.0124	1.0000	-0.5171	-0.4830	-0.2589	0.2841	-0.0124
6	0.3723	0.6413	0.3758	0.2844	-0.5171	1.0000	0.2069	0.4264	-0.2702	0.2844
7	0.5906	0.6538	0.5981	0.5068	-0.4830	0.2069	1.0000	0.6269	-0.5558	0.5068
8	0.9918	0.7660	0.9929	0.9419	-0.2589	0.4264	0.6269	1.0000	-0.8848	0.9419
9	-0.8923	-0.5941	-0.8922	-0.8641	0.2841	-0.2702	-0.5558	-0.8848	1.0000	-0.8641
10	0.9727	0.5674	0.9704	1.0000	-0.0124	0.2844	0.5068	0.9419	-0.8641	1.0000

Correlations FCWEI	1	2	3	4	5	6	7	8	9	10
1	1.0000	0.5720	0.9642	0.9016	0.1002	0.2334	0.4820	0.9821	-0.8923	0.9727
2	0.5720	1.0000	0.5508	0.3936	-0.3593	0.4473	0.6072	0.5874	-0.4847	0.4629
3	0.9642	0.5508	1.0000	0.9568	0.2322	0.1167	0.5622	0.9909	-0.8565	0.9670
4	0.9016	0.3936	0.9568	1.0000	0.4230	-0.0017	0.4517	0.9339	-0.7929	0.9603
5	0.1002	-0.3593	0.2322	0.4230	1.0000	-0.5854	-0.1234	0.1611	0.0112	0.2591
6	0.2334	0.4473	0.1167	-0.0017	-0.5854	1.0000	0.0527	0.1533	-0.1298	0.1361
7	0.4820	0.6072	0.5622	0.4517	-0.1234	0.0527	1.0000	0.5648	-0.4609	0.4183
8	0.9821	0.5874	0.9909	0.9339	0.1611	0.1533	0.5648	1.0000	-0.8747	0.9669
9	-0.8923	-0.4847	-0.8565	-0.7929	0.0112	-0.1298	-0.4609	-0.8747	1.0000	-0.8641
10	0.9727	0.4629	0.9670	0.9603	0.2591	0.1361	0.4183	0.9669	-0.8641	1.0000

b) Mean features correlations of FCBIN (top), and FCWEI (bottom).

TRAIN					Test			
**Best**					**Best**			
	1st	2nd	3rd			1st	2nd	3rd
Connectome model					Connectome model			
SCBIN	[4,6,8]	[4,5,6]	[1,4,6]		SCBIN	[5,6,10]	[2,6,10]	[2,4,6]
SCWEI	[1,3,8]	[3,8,9]	[4,8,10]		SCWEI	[1,3,8]	[3,8,9]	[1,4,8]
FCBIN	[6,8,10]	[4,6,8]	[3,4,5]		FCBIN	[4,6,8]	[1,4,6]	[6,8,10]
FCWEI	[4,5,8]	[1,4,10]	[4,6,8]		FCWEI	[4,5,8]	[3,4,8]	[1,4,8]
**Worst**					**Worst**			
	1st	2nd	3rd			1st	2nd	3rd
Connectome model					Connectome model			
SCBIN	[5,7,9]	[5,6,9]	[4,6,10]		SCBIN	[5,6,9]	[5,7,9]	[4,7,10]
SCWEI	[2,6,7]	[2,5,7]	[1,7,9]		SCWEI	[2,6,7]	[2,5,7]	[1,7,9]
FCBIN	[2,6,7]	[5,7,9]	[5,6,9]		FCBIN	[2,6,7]	[5,7,9]	[5,6,9]
FCWEI	[2,6,7]	[2,7,9]	[2,6,9]		FCWEI	[2,6,7]	[2,7,9]	[2,6,9]

c) 3 best and worst feature combinations.

Supplementary Figure 7: Mean correlation matrices of all nodal features for all connectome models, a) & b), best 3, and worst 3, nodal feature combinations, according to accuracy results, c). Nodal properties nomenclature; 1 = degree, 2 = betweenness, 3 = closeness, 4 = eigenvector, 5 = clustering, 6 = participation, = 7 = within-module degree z-score, 8 = PageRank, 9 =average shortest path, and 10 = subgraph.

Machine learning train and test accuracies for binary structural model in graph spaces ranging from 2 to 10 dimensions on all possible combinations of nodal properties.

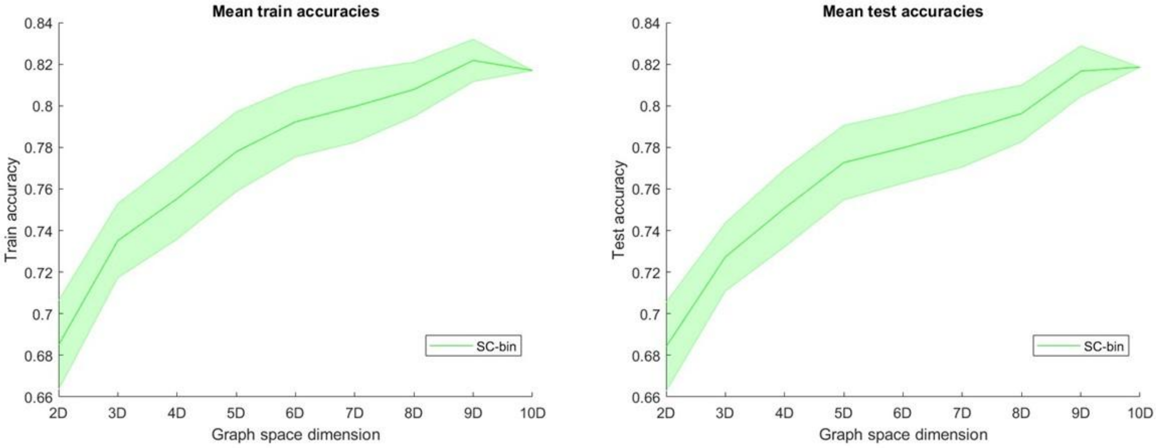

Supplementary Figure 8: Binary structural train and test accuracy curves for every possible combination of nodal features within each dimension. This resulted in a total of 45 classifiers in 2D, 120 in 3D, 210 in 4D, 252 in 5D, 210 in 6D, 120 in 7D, 45 in 8D, 10 in 9D and 1 in 10D, as determined by the combinations possibilities.

Machine learning train and test accuracies for unthresholded weighted structural and functional models in graph spaces ranging from 2 to 10 dimensions.

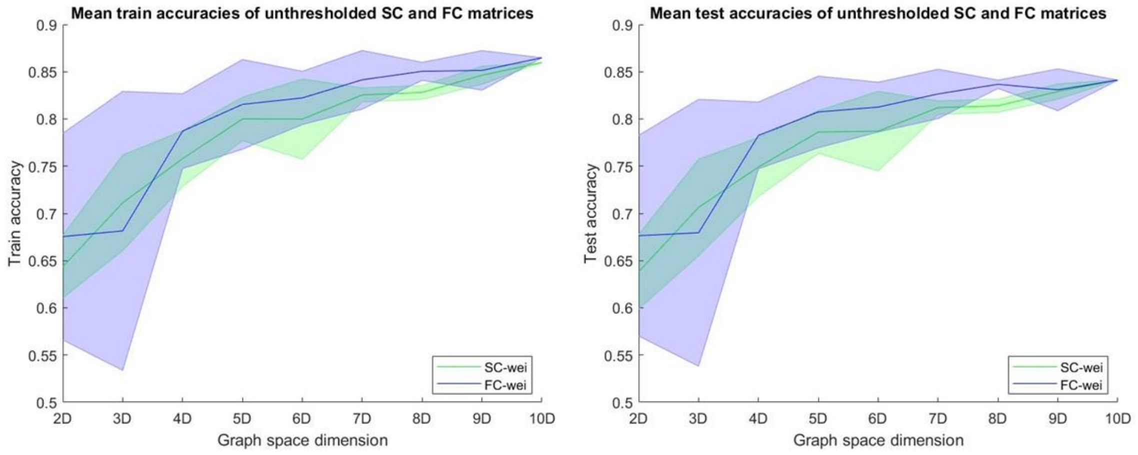

Supplementary Figure 9: Weighted structural and functional ML accuracies on five combinations of nodal features for each dimension. The same 5 combinations of nodal features as for previous ML analysis (Supplementary Table 4) were used to compute the accuracies within each dimension.

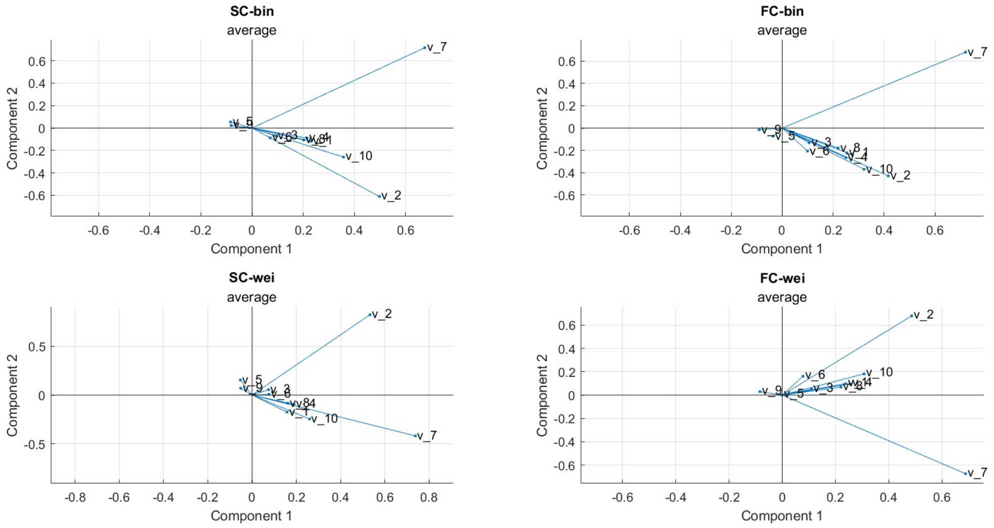

a) 2D latent space from PCA.

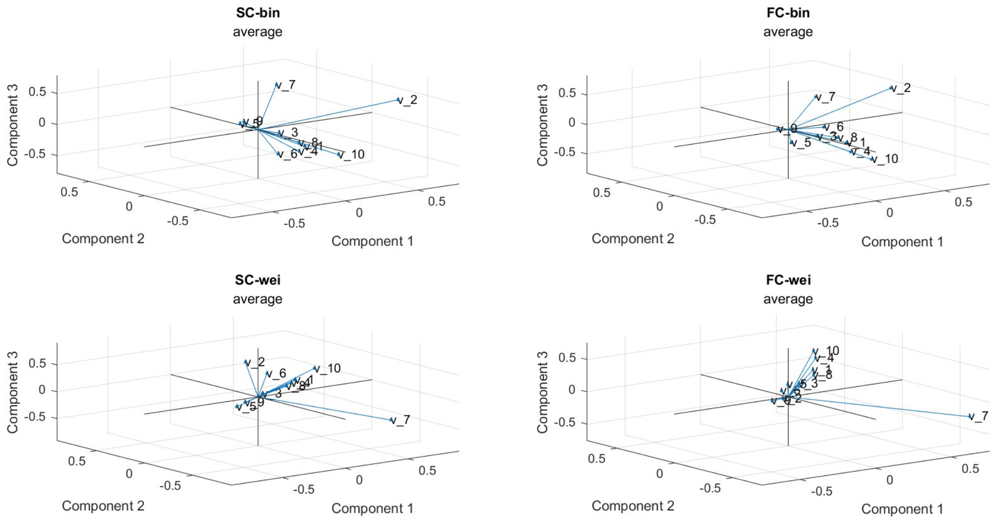

b) 3D latent space from PCA.

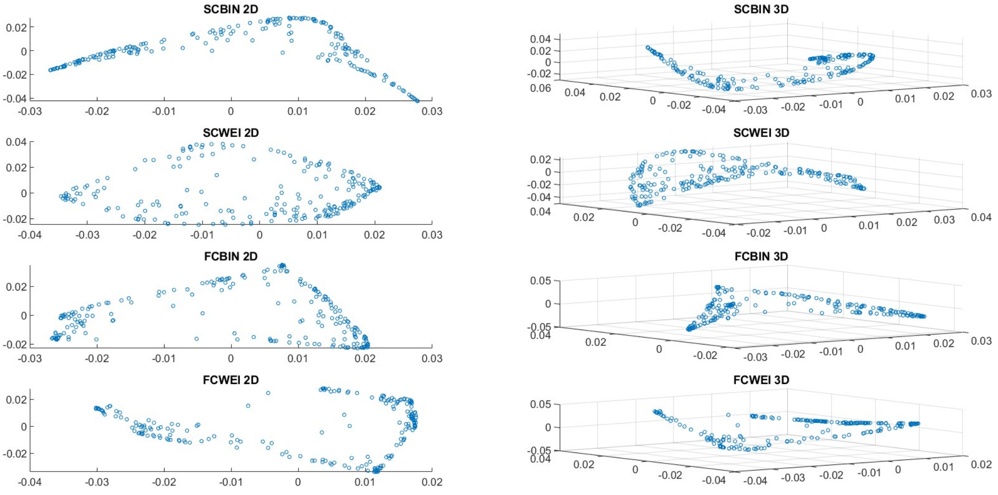

c) 2, and 3D latent space from Laplacian embedding

Supplementary Figure 10: Illustrations of 10D graph spaces embedded into 2 and 3D latent spaces using a Principal Component Analysis (PCA), a) & b), and a Laplacian embedding c). For the PCA, the two, and three, axes correspond to the first two, and three, principal components. Each vector represents one of the 10 nodal features. The direction and length indicate how each feature contributes to the first two and three principal components. For the Laplacian embedding, c), a k-nearest neighbors algorithm creates a network from the 10D graph space data, and the first 3 eigenvectors of the network's Laplacian matrix are the latent space's axis. Each dot represents a brain region projected in the latent space.

Inter-subject nodal distance in 10-dimensional graph spaces.

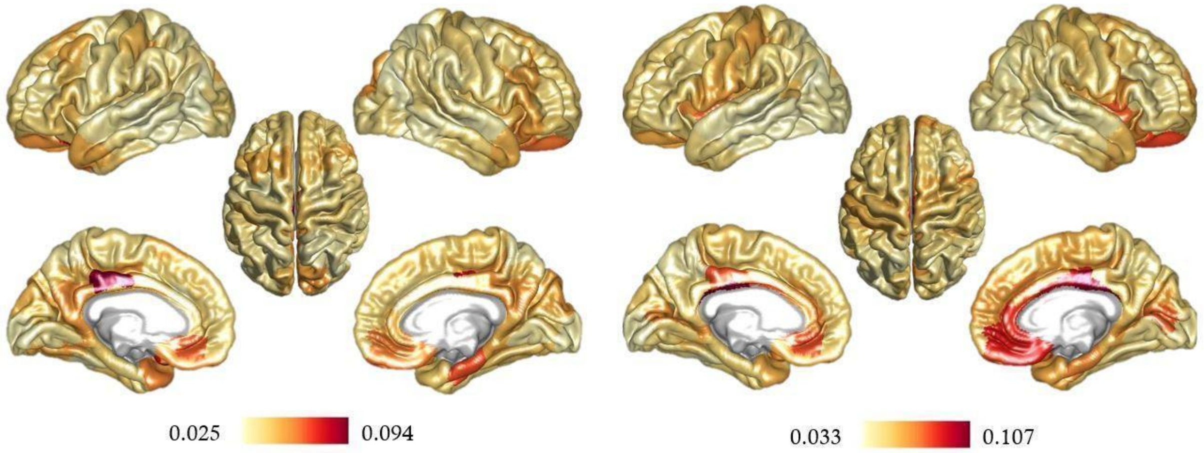

a) Distance of the structural models (SCBIN on the left and Length-SCWEI on the right).

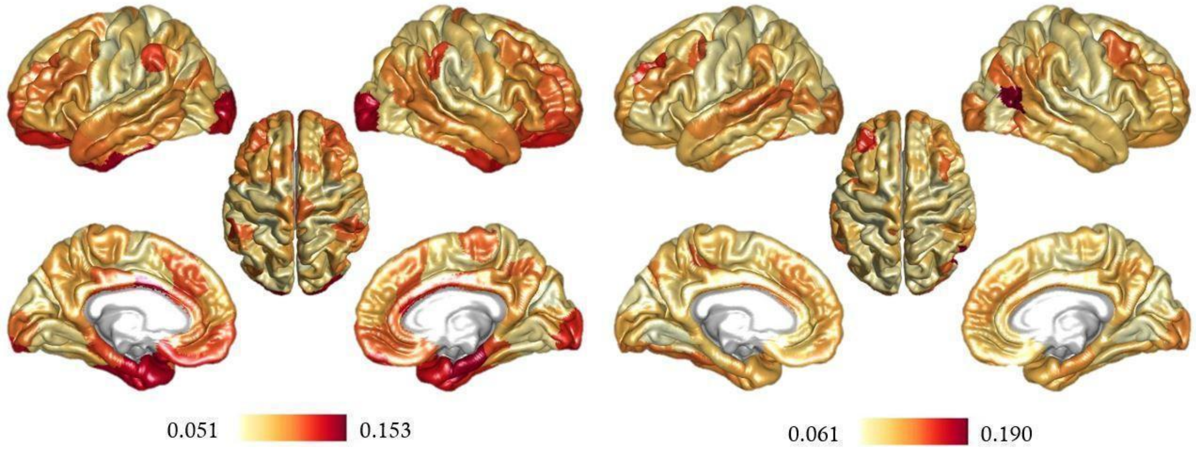

b) Distance of the functional models (FCBIN on the left and FCWEI on the right).

Supplementary Figure 11: Multidimensional single region distance. Brain regions exhibiting a high inter-subject distance are in red, and brain regions showing a low inter-subject distance are in white. These brain maps represent the inter-subject variability from a graph space perspective, with red regions being highly subject-specific and white regions having similar nodal property scores across subjects.

The average distance of a single brain region over all pairs of subjects divided by its standard deviation.

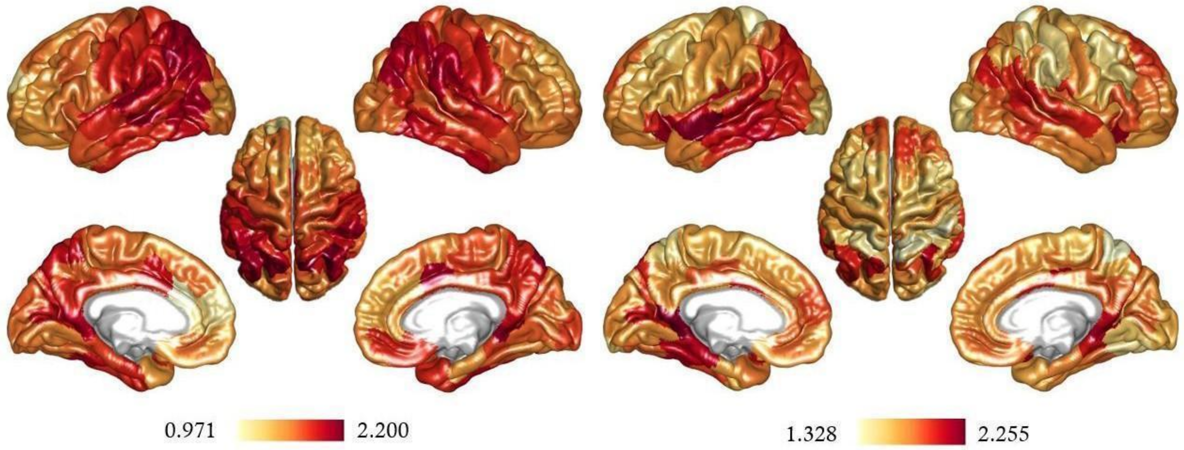

a) Distance of the structural models (SCBIN on the left and Length-SCWEI on the right).

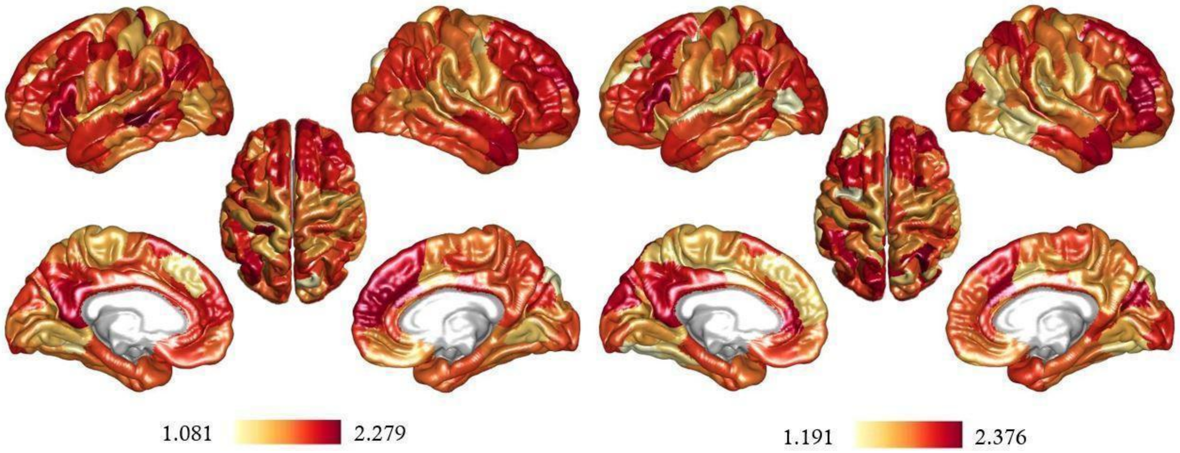

b) Distance of the structural models (FCBIN on the left and FCWEI on the right).

Supplementary Figure 12: Average multidimensional single region distance divided by its standard deviation. Brain regions exhibiting a high distance across every pair of subjects are in red, and brain regions exhibiting a low distance across every pair of subjects are in yellow. These brain maps represent the ‘graph spatial’ network inter-subject variability with red regions being high subject specific brain regions, and white regions being lower subject specific brain regions.

Inter-subject nodal distance in 10-dimensional graph spaces for unthresholded structural and functional models.

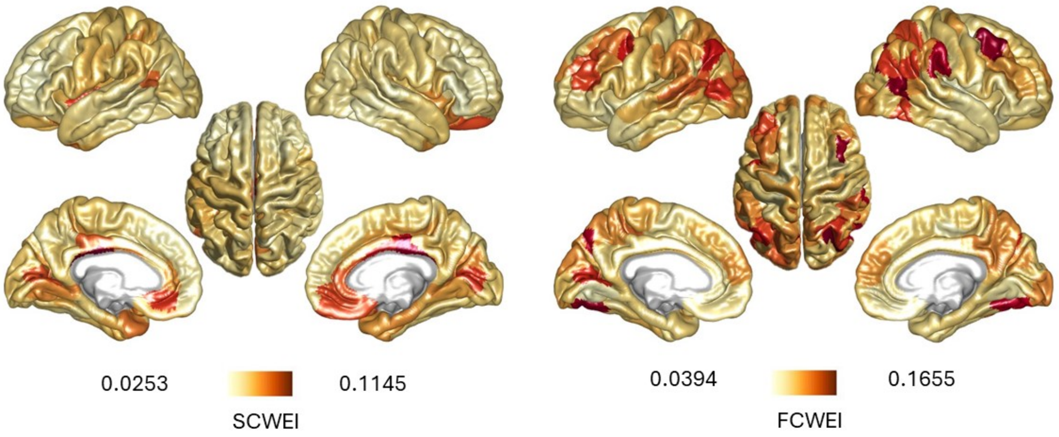

a) Average distance of the unthresholded weighted structural (left) and unthresholded weighted functional (right) model.

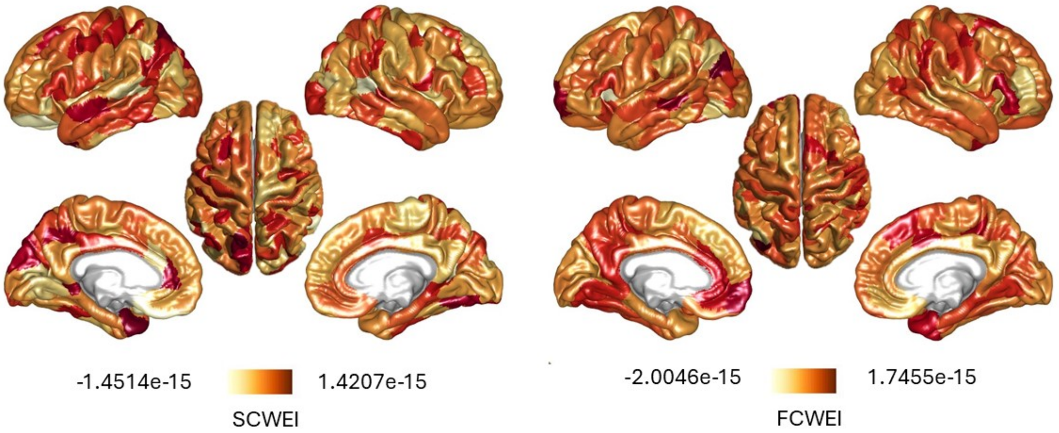

b) Average z-scored distance of the unthresholded weighted structural (left) and unthresholded weighted functional (right) model.

Supplementary Figure 13: Multidimensional single region distance and z-scored distance. Brain regions exhibiting a high distance across every pair of subjects are in red, and brain regions exhibiting a low distance across every pair of subjects are in yellow. These brain maps represent the inter-subject variability from a graph space perspective, with red regions being highly subject-specific and white regions having similar nodal property scores across subjects based on unthresholded networks.

Illustration for a single brain region of both the random and data distributions.

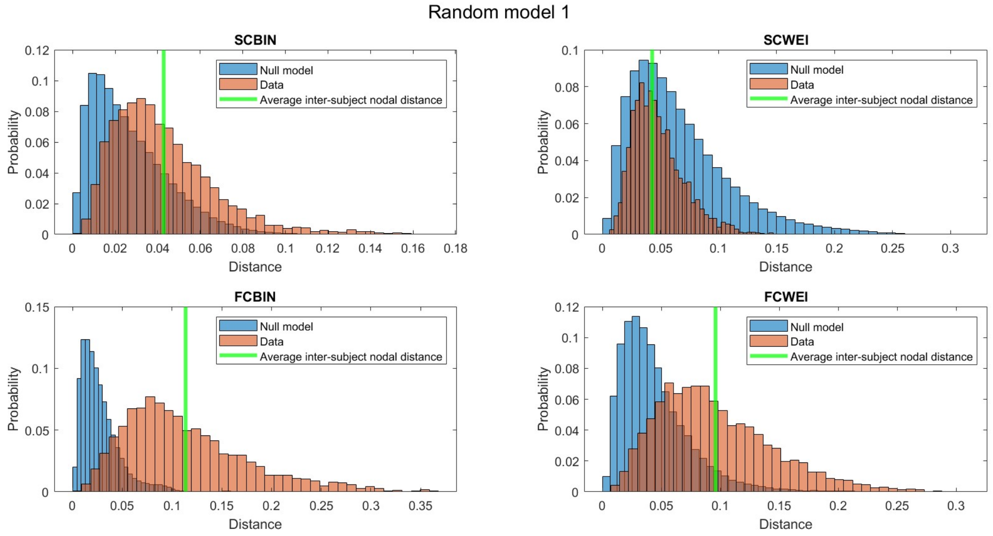

a) Distribution of single region distances of random model 1.

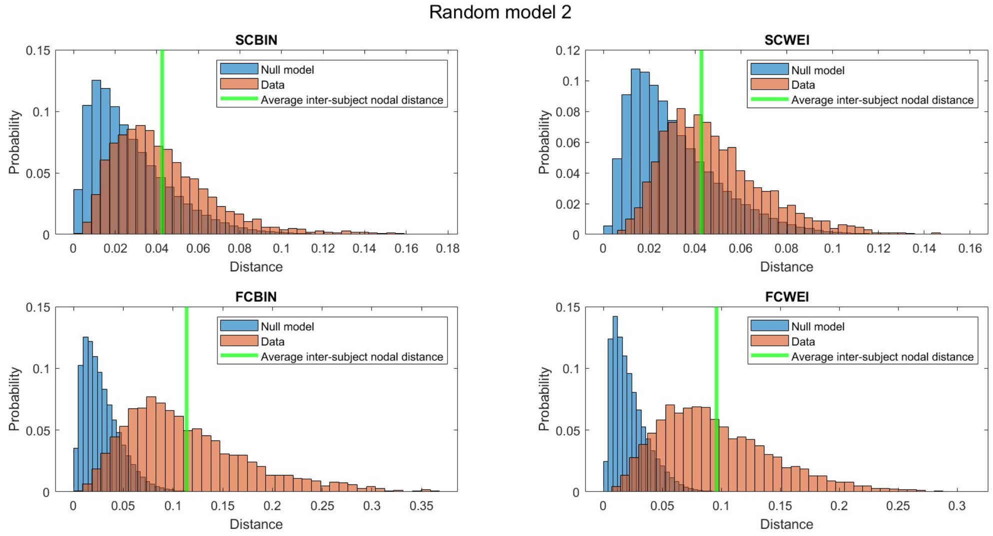

b) Distribution of single region distances of random model 2.

Supplementary Figure 14: Distribution of the 4950 single region distances and the 499500 single region distances resulting from the random model 1, and 2, on the first two rows, and bottom two rows, respectively. Distributions were rescaled to the total number of elements to estimate the probability density function. The green vertical line shows the average inter-subject nodal distance of the HCP data of the same region (average of the single brain region histogram).

Illustration of the significant versus non-significant single region p-values, after FDR correction, from random model 1.

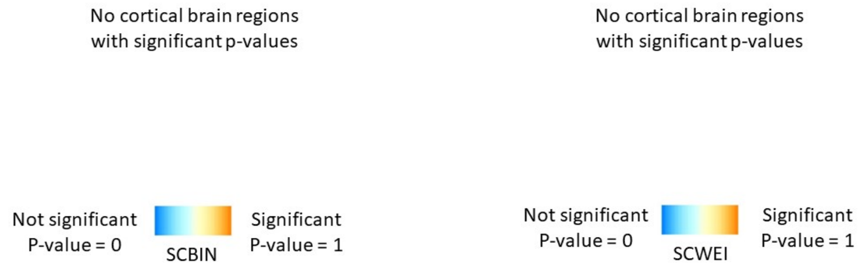

a) P-values of the functional models (SCBIN on the left and Length-SCWEI on the right).

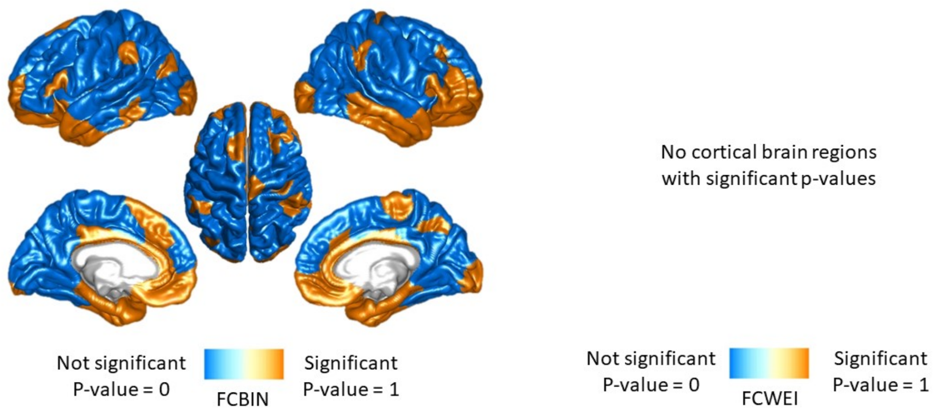

b) P-values of the functional models (FCBIN on the left and FCWEI on the right).

Supplementary Figure 15: Illustration of the FDR corrected p-values resulting from random model 1 on brain maps. The p-values were binarized to 0 (non-significant, color blue) and 1 (significant, color orange) for better visualization.

Illustration of the significant versus non-significant single region after z-score screening from random model 1.

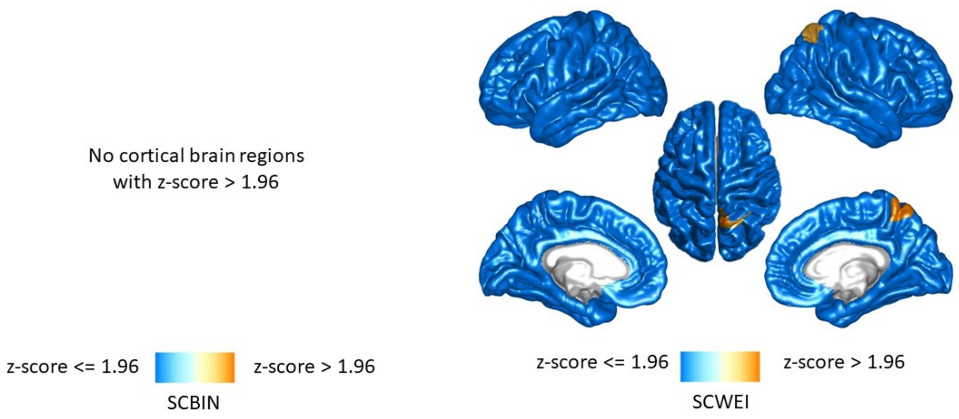

a) Z-scores of the functional models (SCBIN on the left and Length-SCWEI on the right).

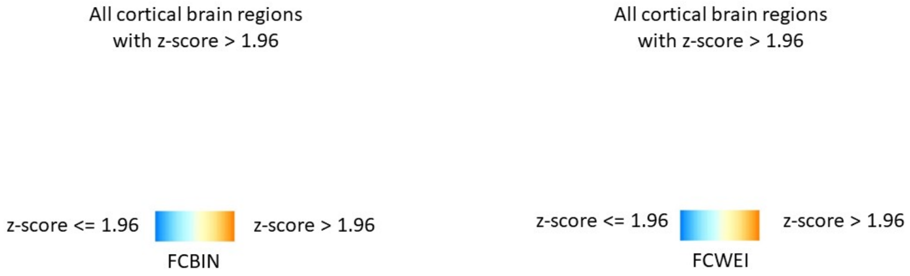

b) Z-scores of the functional models (FCBIN on the left and FCWEI on the right).

Supplementary Figure 16: Illustration of the inter-subject nodal distances, after z-score screening with respect to random model 1, on brain maps. The z-scores were binarized to 0 for z-scores < 1.96 (non-significant, color blue) and 1 for z-scores > 1.96 (significant, color orange) for better visualization.

Illustration of the significant versus non-significant single region after z-score screening from random model 2.

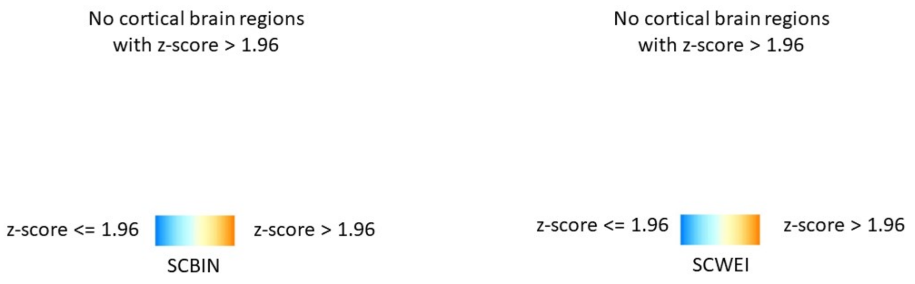

a) Z-scores of the functional models (SCBIN on the left and Length-SCWEI on the right).

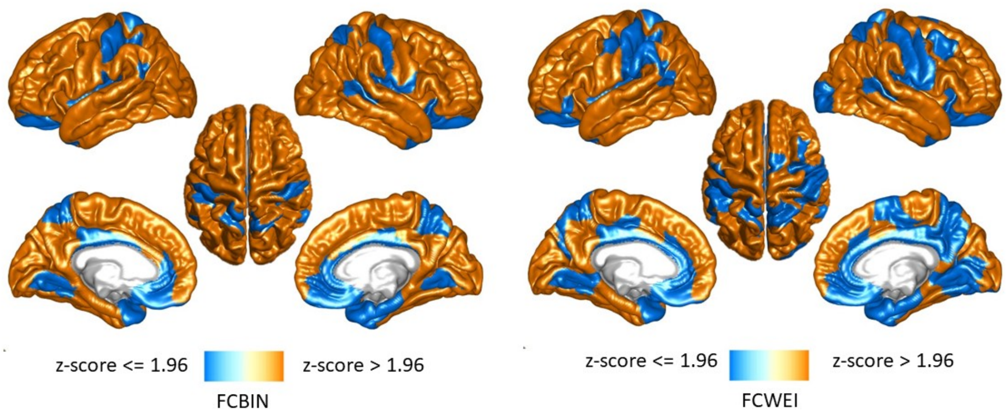

b) Z-scores of the functional models (FCBIN on the left and FCWEI on the right).

Supplementary Figure 17: Illustration of the inter-subject nodal distances, after z-score screening with respect to random model 2, on brain maps. The z-scores were binarized to 0 for z-scores < 1.96 (non-significant, color blue) and 1 for z-scores > 1.96 (significant, color orange) for better visualization.

Global distance between all pairs of subjects at different densities.

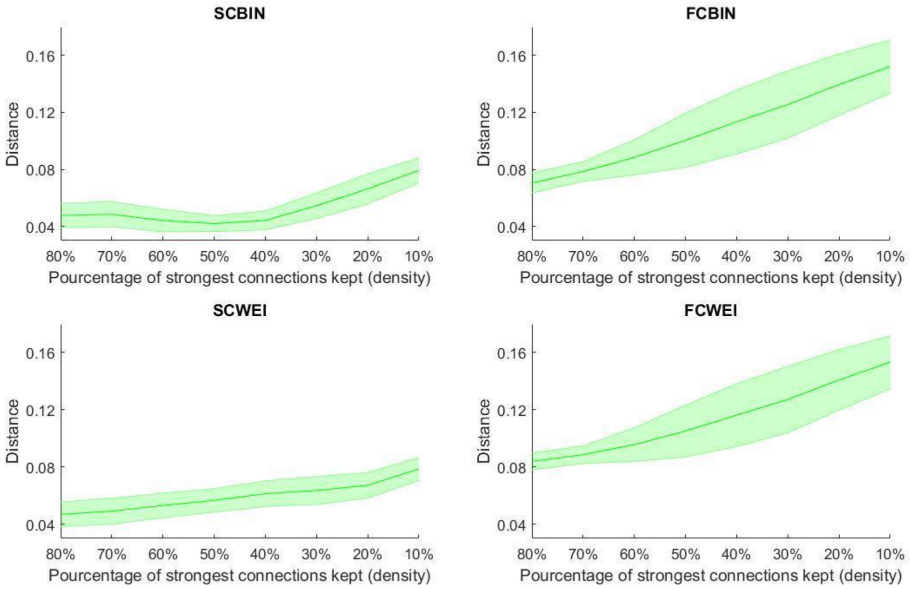

Supplementary Figure 18: Multidimensional global distance between connectomes at different densities with shaded areas representing the standard deviation from the mean.

Global distance between every pair of subjects at each density for every connectome model.

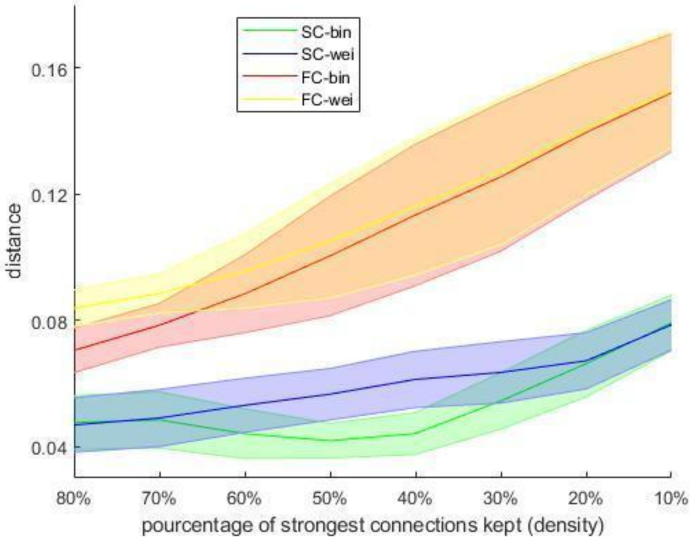

Supplementary Figure 19: Multidimensional global distance between connectomes at different densities. All models together can allow direct comparison, for each density, between the models.
